# Different BI-RADS breast cancer diagnosis using MobileNetV1 and vision transformer based on explainable artificial intelligence (XAI)

**DOI:** 10.1038/s41598-026-37199-2

**Published:** 2026-02-17

**Authors:** Israa Abdelsabour, Ahmed Elgarayhi, Mohammed Sallah, Mohammed Elmogy

**Affiliations:** 1 Physics Department, Faculty of Science, Mansoura University, Mansoura, 35516 Egypt; 2 Information Technology Department, Faculty of Computers and Information, Mansoura University, Mansoura, 35516 Egypt

**Keywords:** Cancer, Computational biology and bioinformatics, Engineering, Health care, Mathematics and computing, Medical research

## Abstract

Breast cancer (BC) remains one of the leading causes of death among women in the world, depending on the requirement for precise, effective, and interpretable computer-aided diagnosis systems (CADs). In this work, a hybrid deep learning (DL) framework is presented for multi-class BI-RADS BC classification using mammographic images. This framework fuses MobileNetV1, a lightweight convolutional neural network (CNN), to capture fine-grained local features and combines it with a Vision Transformer (ViT) to model global contextual connections, thereby enabling corresponding representation learning through a dual-stream structure. The evaluation was performed on the publicly available King Abdulaziz University BC Mammogram Dataset (KAUBC), which includes multi-view mammograms (craniocaudal (CC) and mediolateral oblique (MLO)) arranged according to the BI-RADS classification scheme and characterized by class imbalance. Feature-level fusion is performed, followed by a bagging-based logistic regression (LR) classifier to enhance robustness and decrease prediction conflict. The proposed approach was extensively analyzed using 5-fold cross-validation and compared with multiple state-of-the-art CNN and transformer models, each fused with various machine learning (ML) classifiers. The experimental results demonstrate higher and stable performance across all BI-RADS categories, with accuracy (ACC), sensitivity (SEN), and specificity (SPE) exceeding 99%. In addition, explainable artificial intelligence (XAI) techniques, including Grad-CAM and Grad-CAM++, were applied to provide clinically interpretable visual explanations by highlighting diagnostically relevant regions in mammograms. These results indicate that the proposed MobileNetV1–ViT–Bagging framework recommends an effective, computationally structured, and explainable solution for multi-class BI-RADS BC diagnosis, with strong potential for clinical decision-support applications.

## Introduction

Breast cancer (BC) is the most common cancer among women worldwide, with approximately 2.3 million new cases per year^1^. Despite advances in imaging and pathology, early detection remains challenging due to variations in mammograms, ultrasound (US), and histological (HI) slides, leading to inaccurate classification and delayed diagnosis^2^. A mammogram is a key tool for early detection, as it evaluates tissue density, microcalcifications, and masses to assess cancer risk.

The Breast Imaging Reporting and Data System (BI-RADS) categorizes mammogram findings into seven groups (0–6) and classifies breast density from ACR-A (fatty) to ACR-D (highly dense)^3^. Dense tissue obscures lesions, complicating BI-RADS assignment and increasing prediction errors, particularly under time constraints or limited radiologist experience^4–6^.

Artificial intelligence (AI) refers to computational methods that allow machines to achieve tasks traditionally requiring human intelligence, such as pattern recognition, learning from data, decision-making, and prediction. In healthcare, AI, primarily through machine learning (ML) and deep learning (DL), has emerged as an essential tool for analyzing complex, high-dimensional medical data and supporting clinical decisions. AI has shown considerable commitment in medical imaging, particularly in BC diagnosis. DL models, including convolutional neural networks (CNNs), have become effective at detecting and classifying tumors in mammogram images, enhancing early diagnosis and treatment planning^7^. Moreover, explainable AI (XAI) techniques have improved the interpretability of these models, enabling doctors to understand model decisions and build trust in automated systems^8^. Despite this progress, challenges remain in achieving high ACC across all BI-RADS categories while maintaining computational efficiency and clinical interpretability, prompting the development of hybrid and explainable models for BC classification^9^.

AI, DL, and ML enhance imaging explanation, improving BC classification, ACC, and reproducibility^10,11^. Computer-aided diagnosis (CAD) systems, particularly those using CNNs, automate abnormality detection, segmentation, and classification, achieving performance comparable to that of expert radiologists^12,13^. XAI further increases interpretability and trust^14^. CAD systems can also identify high-risk cases, increase delay times, and improve clinical outcomes; however, challenges remain due to the complexity of mammography and limitations of existing feature-extraction and classification techniques^15^. DL architectures such as AlexNet, GoogleNet, ResNet, MobileNet, and EfficientNet have been employed to enhance BC classification^16^. Ensemble learning, fused multimodal data, imaging, clinical, and molecular biomarkers further enhance predictive ACC. Nevertheless, current CNN–CNN-Transformer fusion frameworks often require end-to-end training of large models, incurring substantial computational cost and limiting interpretability, which contradicts clinical assumptions^17^.

Despite the progress of CNNs and transformer-based models in BC classification, present approaches face several limitations: CNNs efficiently catch local patterns, but miss encode global contextual connections; transformers apply global feature modeling but are computationally large and require huge datasets; and many fusion techniques lack interpretability, limiting clinical acquisition^14^. Unlike existing end-to-end CNN-Transformer hybrid architectures, this study introduces a lightweight, interpretable feature-level fusion framework that combines MobileNetV1 for fine-grained local feature extraction with a Vision Transformer (ViT) for global contextual modeling. The fused features are further reduced using PCA and classified by a bagging logistic regression (LR) classifier to improve robustness, computational efficiency, and clinical interpretability^18^.

Unlike pure benchmarking works, this work focuses on the delineation and validation of the lightweight and interpretable MobileNetV1–ViT framework. The evaluation of multiple CNN and transformer-based fusion is included entirely as a supporting investigation to explain architectural options and classifier selection, rather than as the main contribution of the study. A bagging classifier with LR is applied for classification due to its simplicity, interpretability, and resistance to overfitting. The main contributions of this study are summarized as follows:

- Enhanced Image Quality: Adaptive preprocessing with contrast improvement and noise normalization enhances feature visibility for superior lesion characterization.
- Holistic Feature Representation: Local features extracted by MobileNetV1 are integrated with global features from ViT, allowing effective multi-scale lesion analysis.
- Improved Prediction Performance: The fusion of features and ensemble classification enhances superior ACC, SEP, SEN, precision, and F1-score compared to individual DL models and traditional CAD approaches.
- Computational Efficiency: MobileNetV1 gives lightweight feature extraction, while ViT captures long-range dependencies efficiently, supporting practical computational overhead.
- Robust and Interpretable Classification: Bagging with LR increases variance, stabilizes decision borders, and enhances robustness on the KAUBC dataset.
- XAI: Grad-CAM and Grad-CAM++ highlight clinically relevant regions, improving model transparency and consistency for medical applications.

The paper is structured as follows: Section 1 reviews mammographic image analysis. Section 2 presents the methodology, including preprocessing, feature extraction, fusion, and LR classification. Section 3 details the experimental setup, dataset, metrics, and comparisons with existing CAD systems, including XAI visualization. Section 4 concludes and discusses future research directions.

## Related work

BC image analysis has made significant progress in recent years, with techniques commonly divided into conventional feature-based techniques and DL approaches. Although traditional methods hang on hand-crafted descriptors, DL models automatically learn characteristic features, enhancing detection and classification ACC.

### Transformer-based DL approaches

Ahmed et al.^19^ proposed a MAX-ViT-based DL system with multi-scale feature extraction, a Gated Attention Fusion (GAF) module, and Harris Hawks Optimization (HHO) for feature selection, achieving high performance on the King Abdulaziz University Breast Cancer (KAUBC) dataset (ACC 98.2%). Granted that, effectively, the dependence on complex optimization and an XGBoost classifier increases computational cost and may limit clinical deployment. Abimouloud et al.^20^ compared transformer-based architectures (ViT, CCT, TViT) for binary BC classification, achieving remarkable ACCs (>99%). However, recent studies have further investigated transformer-based and hybrid DL frameworks, demonstrating enhanced global and multi-scale feature representation in medical image analysis^21^. Transformer-based architectures have also shown favorable performance across various medical imaging tasks, prompting their adoption for mammography-based BC diagnosis^22^.

### CNN-based multi-view and ensemble approaches

Nguyen et al.^23^ introduced a multi-view Deep Convolutional Neural Network (DCNN) framework integrated with Light Gradient Boosting Machine (LightGBM) for simultaneous BI-RADS and density evaluation, enhancing F1 scores by 5–10% over single-view approaches. This highlights the importance of multi-view fusion, yet interpretability and generalization to other datasets remain unaddressed. Diwakaran et al.^24^ and Qasrawi et al.^25^ leveraged CNN ensembles and hybrid models with multimodal data, achieving high ACC (up to 98.9%) and fast assumptions. However, these frameworks often lack XAI (XAI) components, decreasing clinical transparency. Hybrid CNN models and multi-view ensembles continue to exhibit robust classification on mammography datasets^26,27^.

### Traditional ML approaches

Lee et al.^28^ applied ML classifiers, decision tree (DT), Support Vector Machine (SVM), k-Nearest Neighbor (kNN) for mammographic breast density classification, illustrating moderate Area Under the Curve (AUC) values (80–81%). While this work asserts the utility of ML in BC evaluation, it is limited to breast density assessment and lacks multi-class BI-RADS classification.

Hamyoon et al.^29^ developed an SVM-based framework for US images that merges five morphometric properties with BI-RADS descriptors. The model achieved an AUC of 88.5%, surpassing experienced radiologists, and highlighted the benefits of fusing morphometric features with BI-RADS descriptors to enhance US-based BC classification reliability.

### XAI and interpretability

Tsai et al.^30^ proposed a fully automated BI-RADS classification into eight classes using a block-based Deep Neural Network. Despite high ACC (94.2%) and AUC (97.2%), model complication and block-based input restrictions may decrease adaptability to various mammogram datasets. Mardones et al.^31^ proposed a DNN for US image analysis, employing You Only Look Once (YOLO) for exact Region Of Interest(ROI) detection and multi-class BI-RADS prediction, attaining Cohen’s kappa scores of 0.58–0.64. This study demonstrated that integrating BI-RADS descriptors improves interpretability, offering a clinically transparent US-based BC classification system.

Shi et al.^32^ proposed QGANet for HI image classification, improving noise robustness via quaternion algebra. While effective, these models were primarily evaluated on HI datasets, and their performance on mammogram images still needs to be investigated. Wani et al.^33^ Integrating AI for BC Classification with Explainable Predictions proposed a hybrid CNN + LightGBM framework that achieved high performance (ACC 98.3%, PRE 98.7%, SEN 98.7%, F1 98.7%) and provided explanations using SHAP at local and global levels. While effective in binary BC classification and enhancing interpretability, the approach does not combine multi-scale feature representations, lacks multi-class BI-RADS assist, and may not generalize to various mammogram datasets. This prompts our MobileNetV1 + ViT hybrid framework, which addresses these gaps by combining local and global features, supporting multi-class classification, and incorporating XAI for robust clinical interpretability.

Saharan et al.^34^ proposed a Hybrid CNN + Random Forest (RF) Scheme for BC Detection with XAI Predictions, a CNN + RF hybrid framework that reaches high predictive performance and provides explanations using SHAP at both local and global levels. While DXAIB improves interpretability and transparency, it primarily addresses binary BC classification, lacks multi-scale feature integration, and does not utilize ensemble strategies, limiting generalization to multi-class BI-RADS scenarios. These gaps motivate our MobileNetV1 + ViT framework, which supports multi-class classification, integrates local and global features, and employs bagging for robust and interpretable BC diagnosis.

The importance of XAI for clinical trust has been progressively highlighted in recent medical imaging studies using Grad-CAM, SHAP, and multimodal explanations^35,36^.

### Summary and gap analysis

In summary, prior works achieve high ACC but have common limitations: many need large annotated datasets, the absence of multi-class BI-RADS classification, produce limited interpretability, or are computationally intensive. These gaps motivate the development of our hybrid MobileNetV1 + ViT framework, which efficiently integrates local and global features, supports multi-class BI-RADS classification, and incorporates XAI for transparent, clinically trustworthy diagnosis. Table 1 summarizes the previous studies of BC diagnosis and classification.

Despite significant advances in mammograms for BC diagnosis, assorted challenges remain. First, many studies rely on private or public datasets that lack diversity or external validation, which limits the generalizability of the models advanced to real-world clinical settings^30,31^. Second, class imbalance and insufficient representation of intermediate BI-RADS categories remain significant limitations, as most studies focus on binary or simple classification tasks, thereby reducing ACC^28^.

Third, the lack of XAI techniques, such as Grad-CAM or SHAP, obstructs interpretability and reduces clinicians’ trust in automated predictions. Fourth, most approaches do not utilize multimodal or multi-view information, rely primarily on single-view mammograms, and ignore other relevant medical data that could improve performance^33^.

Fifth, although CNN-Transformer fusion models show promising results in BC diagnosis, they often depend on end-to-end training of large, complex architectures, increasing computational cost and restricting deployment in resource-constrained environments. Furthermore, these models often produce hard-to-interpret outputs and typically require large datasets, which are difficult to obtain in medical imaging^23,24^.

Recent studies in ensemble and hybrid learning provide methodological innovation for our framework. Bagging and meta-learner ensembles have been shown to reduce variance and improve classification robustness on complex datasets^37,38^. At the same time, dimensionality-depleting techniques such as PCA can enhance classifier efficiency by focusing on relevant features^35,39^. Hybrid CNN-based models with advanced embeddings have demonstrated superior feature representation and ACC^40^, and review studies confirm the overall effectiveness of ensemble techniques for robust prediction^40^. These findings collectively justify integrating MobileNetV1 for feature extraction, ViT embeddings for global context, PCA for dimensionality reduction, and Bagging for final classification in our proposed BC diagnosis framework.

The primary research problem identified in this study is the lack of a cohesive BC diagnosis framework that effectively addresses class imbalance, provides interpretable explanations, and ensures computational efficiency for the KAUBC dataset. The identified limitations necessitate a hybrid approach that optimizes computational efficiency, interpretability, and robust multi-class classification performance within the assessed experimental conditions, as proposed in this study with MobileNetV1 + ViT^41^.

To address this research problem, we sought to improve the model’s performance and reduce dataset-specific bias in the KAUBC dataset for BC diagnosis. The KAUBC dataset comprises a diverse set of mammographic images spanning various BI-RADS categories. To mitigate class imbalance and enhance representation of intermediate classes, extensive data augmentation and class-weighted learning were used to ensure balanced model training and improve recognition of minority classes. Unlike previous studies that focused solely on binary classification tasks, our framework accommodates multi-class BI-RADS classification, providing interpretable insights for the assessed dataset.

Additionally, XAI techniques such as Grad-CAM and Grad-CAM++ were employed to visualize and interpret the proposed model’s decision-making processes, thereby improving transparency and fostering clinical trust. The model integrates complementary feature representations to enhance robustness across all BI-RADS categories, with specific architectures detailed in the Methods section. Recent studies underscore prevailing trends in transformer-, CNN-, and XAI-based BC diagnosis systems, thereby reinforcing the justification for our MobileNetV1 + ViT hybrid framework^42^. Table 1 provides a summary of prior research on the diagnosis and classification of BC.

## Materials and methods

This section describes the proposed DL framework for classifying BC using mammographic images. The proposed framework integrates multiple complementary techniques to address key limitations identified in previous BC diagnostic studies, including class imbalance, limited interpretability, and computational inefficiency. The framework consists of four main steps. Image preprocessing was applied to enhance image quality by adjusting contrast, applying data augmentation, resizing, and normalization. The feature extraction method used MobileNetV1 and the ViT Transformer. MobileNetV1 effectively captures small local features, while ViT models excel in understanding global contextual relationships.

Feature fusion subsequently integrates the extracted representations into a unified descriptor that encompasses both local and global information. Feature reduction and classification involved applying PCA to eliminate duplicate features, while a bagging ensemble classifier improved ACC and contributed to more stable predictions in the KAUBC dataset. To improve model interpretability, XAI techniques, such as Grad-CAM and Grad-CAM++, were used to identify regions the model focused on in the evaluated dataset, highlighting the regions that influenced its decisions.

**Fig. 1:**
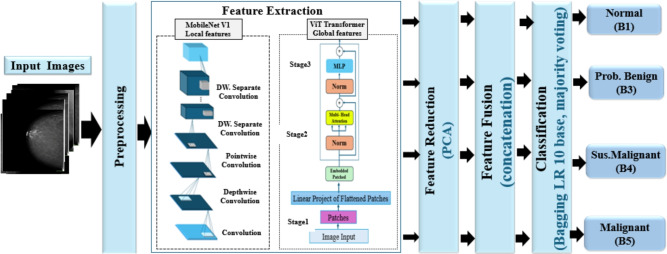
The breast cancer classification framework based on the MobileNetV1 and ViT transformer models.

This can raise radiologists’ trust and improve diagnostic understanding. Figure  1 illustrates the comprehensive structure of the proposed DL framework for BC categorization. This figure illustrates the dual-stream architecture, combining MobileNetV1 for local feature extraction and ViT for capturing global contextual relationships, thereby enhancing feature richness and supporting accurate multi-class BI-RADS classification.

**Table 1:** The comparison of some recent studies.

Study	Dataset	Proposed method	Results
Ahmed et al. (2025)^19^	KAUBCM	Hybrid DL combining MAX-ViT with attention-based fusion (GAFM), HHO feature selection, and XGBoost classifier	ACC: 98.2%, Precision: 98.0%, Recall: 98.1%, F1-score: 98.0%, AUC: 98.9%, MCC: 95.0%
Nguyen et al. (2022)^23^	Internal + DDSM	Multi-view DCNN feature extraction fused with LightGBM classifier	F1-score gains of 5–10% over single-view methods on malignant cases
Lee et al. (2022)^28^	DDSM	ML-based BI-RADS density classification using DT, SVM, and kNN	AUC: 80.1% (DT), 80.5% (SVM), 81.0% (kNN)
Abimouloud et al. (2024)^20^	DDSM	Transformer-based models (ViT, CCT, TViT) integrating convolution and attention	ACC: 99.81% (ViT), 99.92% (CCT), 99.05% (TViT)
Tsai et al. (2022)^30^	Taiwanese Mammograms	DNN with block-based inputs for 8-class BI-RADS classification	ACC: 94.22%, SEN: 95.31%, SPE: 99.15%, AUC: 97.23%
Diwakaran et al. (2023)^24^	MIAS	Transfer learning using Xception and Channel-Boosted CNN (BCP-TL)	ACC: 98.96%
Qasrawi et al. (2024)^25^	20,000 Mammo + 800 clinical	Ensemble model with image enhancement, YOLOv5, and multi-DL classifiers	ACC: 99.7%, Malignant: 98.6%, Benign: 97.2%
Sabani et al. (2022)^43^	7242 Patches / 1744 Mammo	DCNN for soft-tissue BI-RADS classification (opacities only)	ACC: 73.8–89.8%, SEN: 84.0%, SPE: 100.0%
Hamyoon et al. (2023)^29^	1288 US lesions (Malaysia, Iran, Turkey)	SVM with five morphometric features + BI-RADS descriptors	AUC: 88.5%, Radiologist: 81.4%, Resident: 63.2%
Mardones et al. (2022)^31^	749 US nodules	YOLO for ROI detection + DNN for multi-class BI-RADS and malignancy classification	Cohen’s Kappa: 0.58–0.64, high concordance with experts
Wani et al. (2024)^33^	Real-world BC dataset	Hybrid CNN + LightGBM with SHAP explanations at local and global levels	ACC: 98.3%, Precision: 98.7%, SEN: 98.7%, F1: 0.987; Binary classification with improved interpretability

### Image preprocessing

Image preprocessing is a computer-assisted process that modifies digital images to enhance their quality, making them more suitable for subsequent feature extraction stages^44,45^. Mammograms often exhibit noise, low contrast, and class imbalance, which can make feature extraction and classification more challenging^46^. We employ several preprocessing methods to mitigate these problems, including image enhancement, normalization, data augmentation, and resizing, as described in the following paragraphs.

The first preprocessing step is contrast-limited adaptive histogram equalization (CLAHE). It is an advanced preprocessing method that improves local contrast in medical images while reducing the likelihood of noise overamplification^44,45^. CLAHE is notably effective in mammographic imaging for highlighting subtle tissue abnormalities, particularly in areas where traditional global contrast enhancement may be inadequate^47,48^. In contrast to conventional histogram equalization, which modifies pixel intensities across the entire image, CLAHE operates on localized regions, called tiles or windows.

**Fig. 2:**
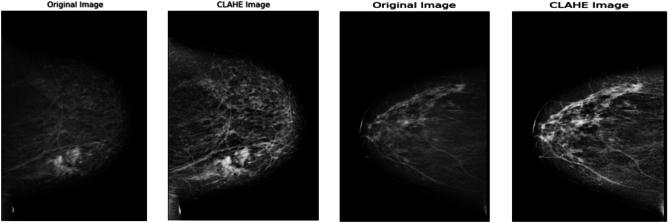
Some sample images from the breast cancer mammogram dataset after applying CLAHE enhancement.

Histogram equalization is applied independently to each tile, and the resulting regions are integrated via bilinear interpolation to produce a seamless final image. CLAHE reduces the excessive amplification of high-frequency noise while preserving local contrast. For mathematical specifics about global histogram equalization, histogram clipping, and mapping functions, refer to Supplementary Material S1. Figure  2 illustrates the impact of CLAHE on mammograms. The graphic illustrates how CLAHE improves local contrast and accentuates tiny features like microcalcifications and lesion borders, which are essential for enhancing the ACC of downstream feature extraction and BC classification.

Using raw mammographic images in a DL model can increase computational complexity and reduce learning efficiency. Image normalization involves adjusting pixel intensities based on statistical metrics. Each pixel value is normalized by subtracting the mean and dividing by the standard deviation (see Supplementary Material S2 for full equation details). This preprocessing method diminishes inter-image variability and accelerates the convergence of DL models, particularly when utilizing transfer learning from pre-trained architectures. By reducing the impact of extraneous brightness and contrast fluctuations, it preserves a uniform distribution of data across the dataset. This stage is crucial for improving the model’s generalization performance in BC classification.

Third, class-wise image data augmentation is an essential method in medical image analysis, particularly when annotated datasets are scarce, as observed in BC classification^18,49^. This study applied geometric transformations to simulate realistic variations in mammogram acquisition and patient positioning. Rotation ($\pm 15^\circ$) simulates minor patient misalignment, horizontal flipping addresses variations in breast orientations, shifting and shearing ($10\%$) replicate small positional discrepancies, and zooming ($10\%$) represents differences in breast size or image scaling. These augmentations enhance dataset diversity while maintaining diagnostically relevant features, thereby improving model generalization and robustness.

All mammogram images were standardized and resized during preprocessing. Subsequently, images were resized to the input dimensions required by the pre-trained MobileNetV1 and ViT models ($224 \times 224$ pixels), in compliance with established pretreatment standards in radiological imaging. Augmentation is performed using TensorFlow’s ImageDataGenerator, recognized for its effectiveness and reproducibility in medical imaging research.

This augmentation method effectively addresses class imbalance, a prevalent issue in medical datasets. It corresponds with scientifically substantiated data-level methodologies to mitigate overfitting and improve model generalization in unbalanced classification issues.

To statistically examine class imbalance, Table 2 presents the count of images and patients per BI-RADS category, indicating that the suspicious and malignant classes are underrepresented. A stratified five-fold cross-validation was employed to maintain class balance across folds. Furthermore, data augmentation was implemented more comprehensively for the minority classes, guaranteeing a more equitable representation during training and improving model generalization across all BI-RADS categories.

A limitation of the preprocessing steps is that the resizing and grayscale conversion of mammographic images may lead to a loss of dynamic range and subtle diagnostic features. Implementing these steps is essential for standardizing input to neural network models; however, they may diminish visibility of fine structures, including microcalcifications and small masses. Future research may investigate adaptive resizing, multi-resolution inputs, or the preservation of original image channels to address these issues.

The preprocessing pipeline includes the application of Contrast Limited Adaptive Histogram Equalization (CLAHE) to enhance local contrast and improve the visibility of subtle mammographic structures, in addition to image resizing to ensure consistent input dimensions for the DL models. While these preprocessing steps improve model convergence and reduce overfitting, it is acknowledged that image resizing may result in a partial loss of dynamic range and attenuation of very fine diagnostic details. However, this effect is mitigated by using contrast enhancement techniques and moderate resizing factors. Future work may explore advanced data augmentation strategies, multi-scale learning approaches, or GAN-based synthetic image generation to further preserve subtle diagnostic cues and enhance model robustness.

Resizing input images to appropriate dimensions is a crucial preprocessing step to ensure compatibility with the input requirements of selected pre-trained models. In this study, MobileNetV1 and the Vision Transformer (ViT) require fixed-size inputs; therefore, mammographic images were resized to 224 $\times$ 224 pixels. Ensuring uniform image dimensions facilitates batch processing and reduces input shape inconsistencies during both training and inference.

Bilinear interpolation was employed during the resizing process to preserve spatial coherence and minimize geometric distortion while maintaining essential structural information. Nevertheless, it is recognized that interpolation-based resizing may slightly affect intensity distribution and very subtle textural patterns in mammograms. Despite this limitation, the adopted resizing strategy provides a practical balance between computational efficiency and preservation of diagnostic information, enabling effective feature extraction and robust learning within the proposed hybrid DL framework for BC diagnosis.

### Feature extraction

**Fig. 3:**
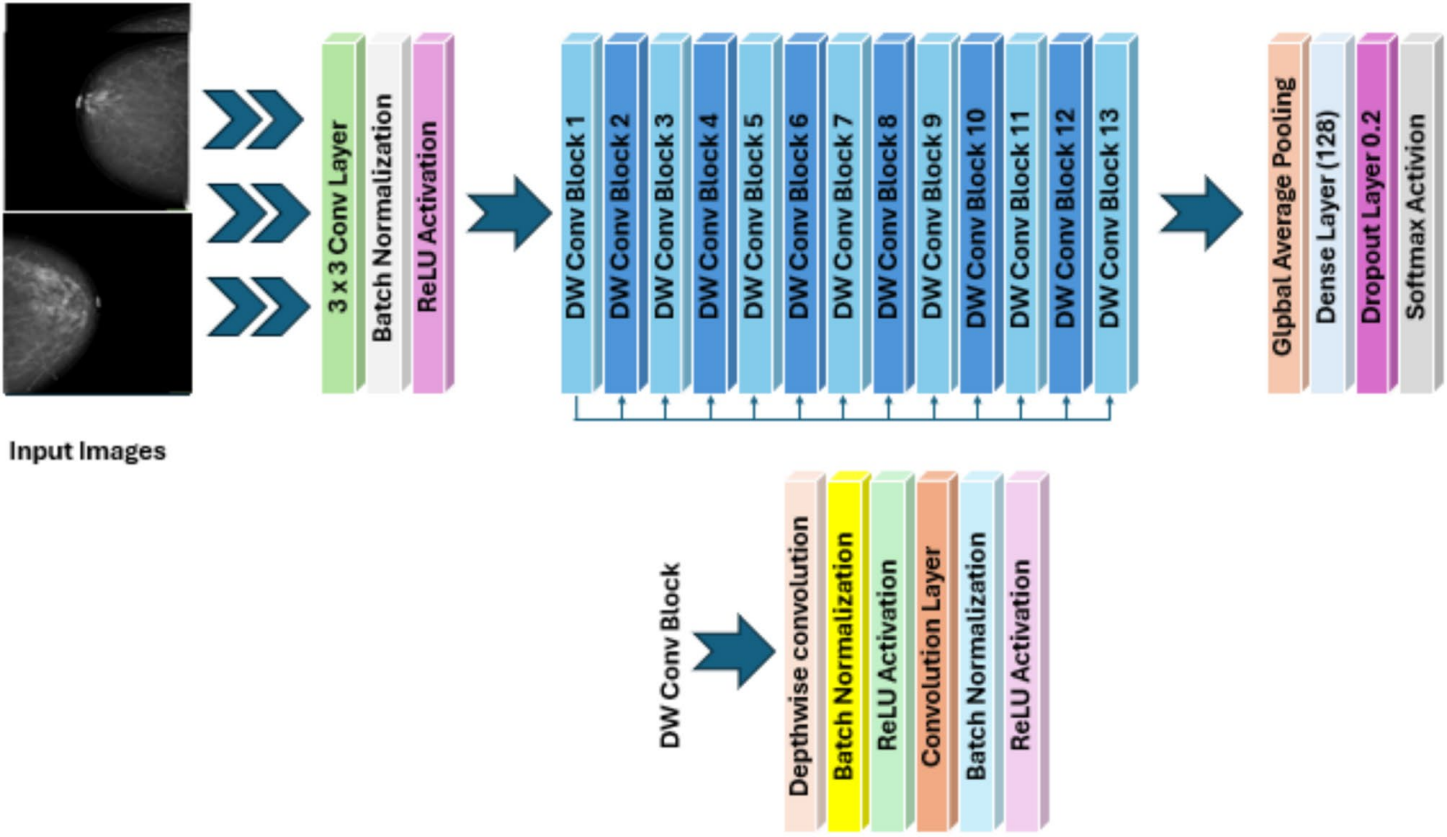
The structure of the MobileNetV1 architecture.

Feature extraction is an essential process for detecting distinguishing patterns that enable discerning between diverse stages and types of BC in mammographic images^50^. The quality of extracted features significantly influences the precision and effectiveness of the classification process. This section outlines the feature-extraction methodology used in the proposed DL system. The proposed method uses MobileNetV1, a lightweight CNN pre-trained on ImageNet, to extract key local features from mammographic images^26^. As illustrated in Fig.  3, MobileNetV1 functions as the main feature extractor, pointing up how its depthwise separable convolutions efficiently capture local texture patterns and fine-grained details such as calcifications and lesion boundaries, making it well-suited for resource-constrained clinical environments^51^.

MobileNetV1 offers several advantages in BC imaging, including computational efficiency, local feature SEN, and suitability for low-resource environments. For detailed mathematical formulations of depthwise convolution, pointwise convolution, and residual connections, see Supplementary Material S3.

These characteristics make MobileNetV1 particularly appropriate as a backbone for mammographic BC analysis, as it combines computational efficiency with valuable extraction of local spatial features crucial for detecting fine-grained patterns like microcalcifications, and lesion contours. The proposed study also appoints a ViT architecture to extract global contextual relationships and high-level semantic features from mammographic images. Unlike CNNs, which operate within limited receptive fields, ViT treats the entire image as a sequence of patches and processes them with a transformer encoder^52^.

**Fig. 4:**
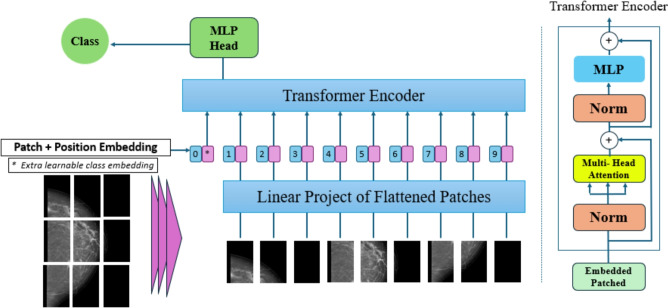
The structure of the ViT transformer used for breast cancer classification. The model processes mammogram images as sequences of patches, each linearly embedded and fed into a transformer encoder to capture long-range dependencies.

This mechanism enables ViT to capture long-range dependencies, crucial for recognizing fine diagnostic patterns in mammographic scans. Figure  4 illustrates that the input image is initially segmented into fixed-size, non-overlapping patches. This figure illustrates how the ViT architecture treats each patch as a distinct token, enabling the model to capture long-range dependencies and global contextual information across the entire mammogram, which is critical for recognizing fine diagnostic patterns such as architectural distortions and dispersed microcalcifications. Each patch is flattened and planed into a latent vector space via a linear embedding layer, with positional encoding and a learnable classification token attached. For full mathematical details of patch embedding, positional encoding, and the self-attention system, see Supplementary Material S4. ViT was selected as a complementary backbone because it captures long-range dependencies and global contextual relationships in mammograms, effectively complementing the local feature extraction of MobileNetV1 and improving overall model performance in multi-class BC classification.

### Fine-tuning

Pre-trained ViT and MobileNetV1 were used as the foundational architectures for feature extraction in the proposed DL framework for BC classification. Using pre-trained models with small, domain-specific datasets, such as mammograms, is beneficial because it allows networks to be initialized with weights from large datasets like ImageNet. This improves generalization^10,18^. A fine-tuning strategy was implemented utilizing various essential techniques to tailor these pre-trained models to the specific attributes of our mammogram dataset.

1. *Freezing of initial layers* The early layers of both MobileNetV1 and ViT were kept frozen, as they capture fundamental, domain-independent visual features such as edges and textures. Preserving these pre-trained weights helps retain useful feature representations learned from ImageNet, thereby reducing overfitting on the KAUBC mammographic dataset.
2. *Progressive unfreezing for domain adaptation* After training the top classification layers, selective lower layers were gradually unfrozen. This progressive unfreezing strategy enables the models to fine-tune incrementally, starting with layers closest to the output and gradually extending deeper into the network. Such a controlled adaptation promotes smoother convergence and enhances the network’s ability to capture domain-specific BC characteristics.
3. *Regularization via Dropout* Dropout layers were incorporated within the final dense layers to help reduce overfitting on the KAUBC dataset. By randomly deactivating a subset of neurons during training, the model is encouraged to learn more diverse feature representations within the dataset under evaluation.
4. *Lower learning rate for controlled adjustment* A smaller learning rate was adopted during the fine-tuning phase to ensure subtle and stable updates to model parameters, preserving valuable pre-trained features while refining them for BC image classification.

This fine-tuning strategy enables MobileNetV1 and ViT to transfer knowledge from large-scale natural image datasets and adapt it to mammographic data, thereby improving classification accuracy and contributing to more stable predictions on the KAUBC dataset.

### Feature reduction with PCA

After feature extraction, PCA is applied to improve generalization and reduce feature dimensionality. PCA projects the high-dimensional feature spaces of ViT and MobileNetV1 onto a lower-dimensional subspace while preserving the maximum variance. To select the number of primary elements, 95% of the growing variance was maintained, ensuring minimal information loss while notably reducing dimensionality and computational cost^35^.

This procedure separates redundant or less instructive assignments, enhancing the signal-to-noise ratio, mitigating overfitting, improving model training efficiency, and decreasing memory running, a crucial factor for clinical implementation. Consequently, PCA yields more compressed and interpretable feature sets by concentrating variance in a smaller number of principal components, thereby improving the effectiveness of the subsequent classifier^39^.

From a clinical perspective, combining ViT and MobileNetV1 yields a powerful, complementary feature-extraction system. MobileNetV1 efficiently extracts local, texture-based patterns using its lightweight depthwise convolutions, making it highly effective at capturing fine-grained details, such as microcalcifications and lesion margins. In parallel, ViT excels at modeling long-range, global dependencies through its self-attention mechanism, thereby integrating contextual information across the entire image, including structural asymmetries and tissue distribution patterns.

Within the proposed hybrid DL framework, this dual approach ensures a comprehensive analysis by capturing both localized details and global context. This synergy contributes significantly to a more accurate, robust, and explainable BC diagnosis, providing a solid foundation for scalable, real-time clinical systems.

### Feature fusion

Feature-level fusion functions are employed at a higher abstraction level than pixel-based techniques, combining extensive semantic information from multiple sources^53^. Our proposed DL framework for multiclass classification employs feature-level fusion to integrate the advantages of MobileNetV1 and the ViT Transformer. After feature extraction, the feature vectors from each model are aggregated to obtain a cohesive representation. The mathematical formulation for the fused feature vector is provided in Supplementary Material S2. The resulting unified feature vector integrates both granular and contextual cues, enhancing multi-class BC classification.

The proposed hybrid framework fuses MobileNetV1 and ViT to support their complementary strengths. MobileNetV1, a lightweight CNN, is productive in capturing fine-grained local features relevant to mammographic analysis. In contrast, ViT employs a self-attention mechanism to capture long-range domination and contextual connections across the entire image. The integration of these two architectures enables the framework to benefit from both precise local feature extraction and global contextual understanding.

While other hybrid models, such as ViT combined with EfficientNet or ResNet, could attain similar performance, MobileNetV1 was selected due to its computational efficiency and suitability for deployment in resource-constrained clinical environments. Previous studies have similarly highlighted the advantage of combining multiple feature extraction techniques and fusion strategies in medical image classification^54^, demonstrating enhanced performance and robustness.

Detecting subtle mammographic features, such as microcalcifications and architectural distortions, is extremely challenging in dense breast tissue or early-stage lesions. The proposed hybrid framework addresses these challenges by combining MobileNetV1, which captures fine-grained local features, with a ViT that models global context and long-range dependencies. Image preprocessing techniques, such as CLAHE, enhance local contrast and improve the visibility of subtle features. Additionally, data augmentation increases model robustness to variations in tissue density and imaging conditions. XAI methods, including Grad-CAM and Grad-CAM++, enable the visualization of regions that influence model predictions, ensuring that clinically relevant, subtle features are effectively considered.

Once the fused features are obtained, they are given into a robust classification stage designed to efficiently handle high-dimensional, multi-class data. The combination of MobileNetV1, ViT, and Bagging LR forms a complementary and synergistic pipeline: MobileNetV1 captures fine-grained local features, ViT models global contextual relationships, and bagging LR combines these features into robust, stable, and interpretable predictions. Together, these components ensure comprehensive feature representation, accurate multi-class BI-RADS classification, and improve model generalization, addressing challenges such as class imbalance and intra-class variability.

### Classification

After integrating feature representations from MobileNetV1 and the ViT Transformer to create a full feature set, the proposed framework proceeds to the classification step using a Bagging ensemble^37^. This design was selected for multiple reasons: the extracted deep features exhibit high discriminative power, enabling a straightforward linear model such as logistic regression to effectively learn class boundaries without increasing network complexity, thereby minimizing the risk of overfitting on moderately sized datasets while delivering well-calibrated probability outputs for ensemble aggregation. Bagging (Bootstrap Aggregating) trains several LR models on distinct bootstrap samples, thereby reducing variance and enhancing stability, which is especially crucial for high-dimensional fused features, where individual logistic regression models may be susceptible to data volatility^38^.

While LR may theoretically manage high-dimensional data, training directly on unprocessed fused features may be computationally demanding; employing PCA for dimensionality reduction allows LR to function effectively by retaining the most useful elements, so improving both performance and efficiency. LR maintains interpretability owing to its linear characteristics, and although bagging marginally diminishes the interpretability of individual models, it greatly enhances resilience and generalization^40^.

Despite LR being linear, the integration of MobileNetV1 and ViT features encapsulates nonlinear interactions, rendering LR appropriate for classifying these deep characteristics. Multiple traditional classifiers, such as SVM and RF, were assessed within the identical fused feature space. LR has consistently demonstrated competitive performance while offering enhanced stability and reduced model complexity, which justifies its inclusion in the proposed framework. Our investigations have shown that Bagging LR achieves performance equivalent to, or superior to, that of softmax or CNN classifiers trained on identical features, underscoring its suitability for medical imaging tasks.

Each base learner predicts class probabilities, and the final decision is determined via majority voting across all LR classifiers. The mathematical formulation for this ensemble method is provided in Supplementary Material S3. The integration of LR with bagging efficiently mitigates intra-class variability, class imbalance, and diverse feature distributions in the KAUBC dataset, yielding a robust and generalizable classifier for BC diagnosis.

## Experimental results

### Dataset description

This research used the KAUBC dataset^41^, a publicly available, real-world clinical dataset designed to enhance BC detection and classification research. The dataset was collected from the Sheikh Mohammed Hussein Al-Amoudi Center of Excellence in BC, affiliated with King Abdulaziz University in Jeddah, Saudi Arabia, and spans the period from April 2019 to March 2020. The KAUBC dataset contains more than 6,000 digital mammograms from 1,416 patients, spanning various breast conditions and BI-RADS assessment categories.

The images were labeled and verified by experienced radiologists according to clinical reports and the standardized BI-RADS lexicon, ensuring reliable ground truth annotations and facilitating the development and evaluation of CAD systems with high ACC. Each patient’s record contains bilateral 2D mammograms captured in two standard views: CC and MLO for each breast. The images are provided in DICOM format, preserving high spatial resolution and diagnostic detail for comprehensive analysis using DL techniques.

The KAUBC dataset comprises authentic clinical screening data, as opposed to synthetic or simulated images. All mammograms underwent anonymization before release, with no personally identifiable information included, thereby ensuring adherence to ethical standards and patient privacy regulations. Ethical approval was secured from the originating institution during data collection, and the dataset is provided exclusively for research purposes. The KAUBC dataset comprises 1,416 female patients aged 25-75 years, with a mean age of 48.3 years. The distribution of patients by age group is as follows: 212 patients (15%) are under 40 years of age, 906 patients (64%) are between 40 and 60 years of age, and 298 patients (21%) are over 60 years of age. All patients originate from the Jeddah region of Saudi Arabia, potentially introducing geographic and demographic bias in the dataset.

**Table 2:** The description of the KAUBC dataset.

BI-RADS category	Number of images	Number of cases	Age range (mean)	Breast density distribution (%)
0 (Incomplete)	440	110	30–80 (55)	10% Low, 30% Mild, 40% Moderate, 20% High
1 (Normal)	1884	471	30–79 (52)	5% Low, 25% Mild, 50% Moderate, 20% High
2 (Benign)	3250	812	32–77 (50)	4% Low, 28% Mild, 47% Moderate, 21% High
3 (Probably Benign)	387	90	34–78 (53)	6% Low, 24% Mild, 46% Moderate, 24% High
4 (Suspicious)	102	26	35–76 (56)	2% Low, 15% Mild, 50% Moderate, 33% High
5 (Malignant)	24	6	38–72 (58)	0% Low, 10% Mild, 40% Moderate, 50% High

To mitigate information leakage and guarantee an unbiased evaluation, the KAUBC dataset was divided at the patient level into training and test sets, adhering to an 80–20 ratio. All mammograms from an individual patient were assigned to a single subset, ensuring that images from the same patient were not present in both the training and test sets. This patient-level splitting mitigates the risk of overoptimistic performance estimates that may occur if the model encounters similar patterns from the same patient during training.

Compliance with this procedure was verified through programming, confirming that patient identifiers in the training and test sets are disjoint, thereby ensuring a clinically meaningful assessment of the proposed MobileNetV1–ViT framework. KAUBC, like many real-world medical imaging datasets, exhibits class imbalance across BI-RADS categories, especially for suspicious and malignant cases. A stratified five-fold cross-validation strategy was utilized to ensure reliable evaluation by preserving representative class distributions across all folds. Table  2 presents the dataset characteristics. Our study focused on images categorized as BI-RADS 1, 3, 4, and 5, which denote normal, probably benign, suspicious for malignancy, and malignant findings, respectively.

**Fig. 5:**
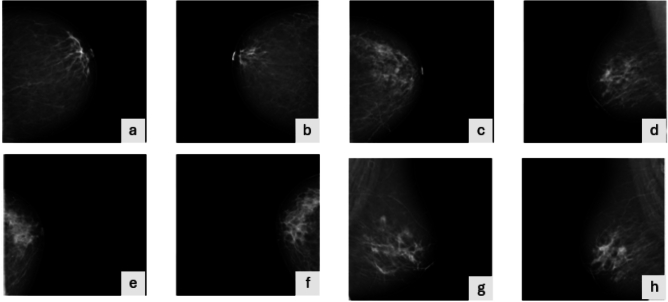
Examples representing the four BI-RADS categories included in the KAUBC dataset: (**a**,**b**) Normal, (**c**,**d**) Malignant, (**e**,**f**) Probably Benign, and (**g**,**h**) Suspicious Malignant in both CC&MLO image views.

Figure 5 illustrates representative sample images from the KAUBC dataset. This figure highlights the diversity of mammographic appearances across BI-RADS categories, demonstrating variations in tissue density, lesion size, contrast, and morphology. These variations underscore the challenges inherent in multi-class BC classification and underscore the need for robust, discriminative feature extraction strategies.

### Performance evaluation

To thoroughly assess the performance of the proposed DL model for multi-class BC classification, a set of evaluation metrics was employed, presented in Eqs. (1)–(8). These metrics provide a comprehensive view of the model’s effectiveness, capturing overall ACC, the ability to correctly identify positive and negative cases, and consistency across classes. The key metrics include:

- *ACC* The ratio of correctly classified cases to the total number of cases^55^.
  1 $$\begin{aligned} \text {ACC} = \frac{TP + TN}{TP + TN + FP + FN} \end{aligned}$$
  where TP (True Positives) and TN (True Negatives) denote accurately classified cases, whereas FP (False Positives) and FN signify misclassified examples.
- *SEN / Recall* The proportion of TP cases correctly identified^55^.
  2 $$\begin{aligned} \text {SEN} = \frac{TP}{TP + FN} \end{aligned}$$
- *SPE* The proportion of TN cases correctly identified^55^:
  3 $$\begin{aligned} \text {SPE} = \frac{TN}{TN + FP} \end{aligned}$$
- *Precision* The fraction of predicted positive cases that are actually positive^55^:
  4 $$\begin{aligned} \text {Precision} = \frac{TP}{TP + FP} \end{aligned}$$
- *F1-Score* The harmonic mean of precision and SEN^55^:
  5 $$\begin{aligned} \text {F1-Score} = \frac{2 \times \text {Precision} \times \text {SEN}}{\text {Precision} + \text {SEN}} \end{aligned}$$
- *Receiver Operating Characteristic (ROC) Curve* Plots the true positive rate (TPR) against the false positive rate (FPR), which is $1 - \text {TNR}$. It is widely used in radiology to evaluate diagnostic performance^56^:
  6 $$\begin{aligned} \text {TPR} = \frac{TP}{TP + FN}, \quad \text {FPR} = \frac{FP}{FP + TN} \end{aligned}$$
- *Area Under the ROC Curve (AUC)* Quantifies the classifier’s ability to distinguish between classes. Higher AUC indicates better discrimination^57^:
  7 $$\begin{aligned} \text {AUC} = \int _{0}^{1} \text {TPR}(x) \, d\text {FPR}(x) \end{aligned}$$
- *Balanced Accuracy (BACC)* Addresses class imbalance by averaging SEN and SPE across all classes^55^:
  8 $$\begin{aligned} \text {BACC} = \frac{\text {SEN} + \text {SPE}}{2} \end{aligned}$$

The use of these metrics ensures a holistic evaluation of the proposed DL model. This multi-metric assessment provides critical insights into the model’s strengths and limitations, particularly in BC classification, where high SEN and SPE are essential for reliable diagnosis and treatment planning.

### Results

In this section, we present the experimental results of the proposed framework and compare its performance with that of a range of DL architectures, hybrid CNN+ViT models, and traditional classifiers. Furthermore, we analyze the impact of the proposed framework’s components by evaluating the standalone performance of MobileNetV1, its performance with feature reduction and ensemble classifiers, and the effects of various optimization strategies on overall ACC and computational efficiency.

The computational complexity of the proposed framework was assessed on a personal workstation utilizing Anaconda Python 3.1, equipped with an Intel Core i5 CPU (4–6 cores, 2.4–3 GHz) and 16 GB of RAM. MobileNetV1 is a lightweight convolutional neural network (CNN) comprising approximately 3.2 million parameters and 0.57 GFLOPs, facilitating efficient local feature extraction in resource-constrained clinical settings. ViT-B/16, with over 86 million parameters and 17.6 GFLOPs, effectively captures global contextual information in mammographic images. The Swin Transformer (Swin-Tiny), comprising 28 million parameters and 4.5 GFLOPs, employs hierarchical attention to balance local and global feature modeling while maintaining a moderate computational cost.

Feature extraction is conducted independently utilizing pre-trained MobileNetV1, ViT, and Swin models, followed by feature-level fusion and bagging ensemble classification. This method circumvents end-to-end joint training, thereby decreasing computational demands while utilizing complementary local, global, and hierarchical representations.

Average inference latency was assessed on the test set at full image resolution, and memory usage was tracked, validating the framework’s appropriateness for practical clinical applications. Table 3 summarizes the computational analysis, detailing parameters, FLOPs, model size, inference time, and memory usage for each component, thereby demonstrating the framework’s efficiency and usability in practical applications. The proposed hybrid framework exhibits moderate computational complexity, leveraging ViT’s representational capabilities while avoiding end-to-end joint training. Feature-level fusion and Bagging ensemble classification incur negligible additional computational overhead relative to fully integrated deep architectures.

**Table 3:** The estimated computational efficiency of the proposed hybrid framework components, including MobileNetV1, ViT-B/16, Swin Transformer Tiny, and the proposed hybrid model. Metrics include number of parameters, floating point operations (FLOPs), model size, inference time, and memory footprint based on experiments on a personal workstation with Intel Core i5 CPU and 16 GB RAM.

Model	Params (M)	FLOPs (G)	Size (MB)	Inference (ms/img)	Remarks / Memory (MB)
MobileNetV1	3.2	0.57	12	50–70	Lightweight CNN; approximate memory: 50–60 MB
ViT-B/16	86	17.6	330	400–500	Global feature modeling; approximate memory: 1200–1300 MB
Swin-T (Tiny)	28	4.5	90	150–200	Hierarchical local-global attention; approximate memory: 400–450 MB
Proposed Hybrid	89	18.2	342	450–550	Feature-level fusion; no end-to-end training; approximate memory: 1250–1350 MB

We performed an extensive evaluation process to determine the most effective DL methodology for BC diagnosis. Several pre-trained CNN architectures, including EfficientNetB0, DenseNet121, InceptionV3, MobileNetV1/V2, InceptionResNetV2, and VGG16/VGG19, were systematically compared using the KAUBC dataset to identify the optimal feature extractor for accurate BC classification. To mitigate overfitting and guarantee reliable performance assessment, a 5-fold cross-validation strategy was utilized during the experimental procedure. The objective was to assess the proposed framework against selected existing methods and to evaluate its diagnostic performance using the KAUBC dataset. Various regularization techniques, including dropout and early stopping, were used during model training. Dropout was applied at multiple network layers to mitigate overfitting associated with specific neuron activations.

Simultaneously, early stopping was implemented to terminate training when validation performance ceased to improve, thus enhancing generalization to unseen data. The 5-fold cross-validation implementation reinforced this objective by minimizing variance and improving model generalization. Among the pre-trained models, MobileNetV1 achieved the highest independent ACC (97%), followed by VGG16 (96.9%) and MobileNetV2 (96.4%), indicating that lightweight architectures enhance BC detection. Table 4 provides an evaluation of pretrained models, outlining their effectiveness in BC classification.

After identifying the most optimistic pre-trained models, we conducted a systematic investigation to examine how different classifiers affected overall diagnostic performance for BC detection. Different ML classifiers were applied, like DT, RF, KNN, Gradient Boosting (GB), LR, and SVMs with various kernels (linear, radial basis function (RBF), and polynomial). Each classifier was paired with the extracted features from the pretrained models to evaluate their combined performance and determine whether any classifier could further enhance the models’ discriminative capability. The SVM achieved the highest classification accuracy with the RBF kernel (94.7%), MobileNetV1 (93.1%), and DenseNet121 (93.1%).

**Table 4:** The performance comparison of various pre-trained models for breast cancer classification.

Model	PRE (%)	SEN (%)	SPE (%)	F1-Score (%)	ACC (%)	BACC (%)
EfficientNetB0	90.5	91.0	95.2	91.0	90.8	93.1
DenseNet121	95.5	94.7	98.2	96.0	94.7	96.5
InceptionV3	94.5	94.0	98.0	95.0	93.9	96.0
**MobileNetV1**	**97.7**	**97.1**	**99.0**	**97.2**	**97.0**	**98.1**
MobileNetV2	97.0	94.4	98.8	97.0	96.4	96.6
InceptionResNetV2	95.0	94.5	98.1	95.0	94.4	96.3
VGG16	97.2	96.9	98.9	97.2	96.9	97.9
VGG19	95.2	94.6	98.2	95.5	94.6	96.4

The detailed results of all pre-trained models integrated with different classifiers are presented in Table 5, which indicates that the selection of the classifier significantly influenced the overall classification ACC. Some models, such as MobileNet, exhibit improved performance when combined with specific classifiers, such as SVMs, highlighting the synergistic interaction between feature extractors and classifiers. These findings highlight the importance of investigating the interaction between pre-trained models and classifier selection to achieve the maximum diagnostic ACC in BC diagnosis.

In the next phase, we examined the chance of merging Swin transformers with the most effective pre-trained CNN models identified in the earlier experiments. We hypothesized that the Swin Transformer’s ability to capture long-range dependencies in mammographic images could further enhance BC detection ACC. All ensemble models were evaluated under the same 5-fold cross-validation protocol to ensure consistent and unbiased performance comparison. Each ensemble model, formed by combining a pretrained CNN with Swin, was evaluated using the same set of classifiers applied previously to determine which classifier best leveraged the complementary strengths of both architectures.

**Table 5:** The performance comparison of various pre-trained models with different classifiers for breast cancer classification.

Model	Classifier	PRE (%)	SEN (%)	SPE (%)	F1-Score (%)	ACC (%)	BACC (%)
EfficientNetB0	SVM (Linear)	90.3	90.2	96.3	90.2	90.2	93.3
	SVM (Poly)	89.3	88.7	95.7	88.8	88.7	92.2
	SVM (RBF)	90.2	89.8	96.1	89.8	89.3	92.9
	DT	73.1	73.0	90.2	73.0	73.0	81.6
	RF	86.5	86.1	94.8	86.2	86.1	90.5
	MLP	88.3	81.2	95.3	80.2	80.2	88.3
	KNN	83.7	82.8	93.8	83.0	82.8	88.3
	GB	85.5	85.1	94.4	85.2	85.1	89.8
	LR	86.9	86.6	95.0	86.6	86.6	90.8
DenseNet121	SVM (Linear)	91.5	91.4	96.8	91.4	91.4	94.1
	SVM (Poly)	93.1	92.8	97.2	92.8	92.8	95.0
	SVM (RBF)	93.3	93.1	97.3	93.0	93.1	95.2
	DT	75.9	75.9	91.3	75.9	75.9	83.6
	RF	90.0	90.0	96.2	90.0	90.0	93.1
	MLP	93.0	93.0	97.4	93.0	93.0	95.2
	KNN	93.0	93.0	97.0	93.0	93.0	95.0
	GB	89.3	89.1	95.9	89.1	89.1	92.5
	LR	90.8	90.7	96.5	90.7	90.7	93.6
InceptionV3	SVM (Linear)	89.4	89.3	96.0	89.2	89.3	92.7
	SVM (Poly)	91.0	90.5	96.3	90.5	90.5	93.4
	SVM (RBF)	91.8	91.5	96.7	91.5	91.5	94.1
	DT	74.6	74.5	90.9	74.6	74.5	82.7
	RF	88.8	88.3	95.5	88.3	88.3	91.9
	MLP	90.6	90.6	96.6	90.6	90.6	93.6
	KNN	86.3	85.3	94.8	85.3	85.3	90.0
	GB	88.0	87.7	95.3	87.7	87.7	91.5
	LR	88.8	88.6	95.7	88.6	88.6	92.2
MobileNetV1	SVM (Linear)	93.7	93.6	97.6	93.6	93.6	95.6
	SVM (Poly)	94.8	94.6	97.8	94.5	94.6	96.2
	SVM (RBF)	94.0	94.7	97.9	94.6	94.7	96.3
	DT	80.3	80.2	92.8	80.2	80.2	86.5
	RF	92.3	92.0	96.9	92.0	92.0	94.5
	MLP	93.9	93.9	97.8	93.9	93.9	95.9
	KNN	88.0	87.2	95.4	87.2	87.2	91.3
	GB	91.7	91.5	96.7	91.5	91.5	94.1
	LR	92.9	92.7	97.2	92.7	92.7	95.0

Model	Classifier	PRE (%)	SEN (%)	SPE (%)	F1-Score (%)	ACC (%)	BACC (%)
MobileNetV2	SVM (Linear)	94.2	94.2	97.8	94.1	94.2	96.0
	SVM (Poly)	95.0	94.8	98.0	94.8	94.8	96.4
	SVM (RBF)	95.1	95.0	98.0	94.9	95.0	96.5
	DT	76.6	76.6	92.6	76.6	79.6	84.6
	RF	92.4	92.1	96.9	92.1	92.1	94.5
	MLP	93.8	93.8	97.7	93.7	93.8	95.8
	KNN	90.1	89.6	96.3	89.6	89.6	93.0
	GB	91.5	91.3	96.7	91.3	91.3	94.0
	LR	93.3	93.2	97.4	93.2	93.2	95.3
InceptionResNetV2	SVM (Linear)	88.1	87.9	95.5	87.9	87.9	91.7
	SVM (Poly)	89.9	89.9	96.0	89.9	89.5	93.0
	SVM (RBF)	90.9	90.5	96.4	90.5	90.5	93.45
	DT	70.8	70.8	89.5	70.8	70.8	80.15
	RF	87.4	86.9	95.0	86.9	86.9	90.95
	MLP	89.9	89.9	96.3	89.9	89.9	93.1
	KNN	84.0	82.5	93.8	82.6	82.5	88.15
	GB	85.9	85.5	94.5	85.5	85.5	90.0
	LR	87.0	86.7	95.0	86.8	86.7	90.85
VGG16	SVM (Linear)	87.6	87.4	95.3	87.5	87.4	91.35
	SVM (Poly)	88.8	88.0	95.3	88.1	88.0	91.65
	SVM (RBF)	91.0	90.7	96.5	90.7	90.7	93.6
	DT	74.8	74.7	90.9	74.7	74.7	82.8
	RF	87.5	87.0	95.1	87.1	87.0	91.05
	MLP	88.5	88.5	95.8	88.5	88.5	92.15
	KNN	84.2	82.9	93.9	83.0	82.9	88.4
	GB	85.9	85.5	94.5	85.6	85.5	90.0
	LR	86.8	86.5	94.9	86.5	86.5	90.7
VGG19	SVM (Linear)	87.4	87.2	95.2	87.2	87.2	91.2
	SVM (Poly)	88.7	87.9	95.3	88.0	87.9	91.6
	SVM (RBF)	90.9	90.5	96.4	90.6	90.5	93.45
	DT	76.5	76.4	91.5	76.4	76.4	83.95
	RF	87.0	86.5	94.9	86.6	86.5	90.7
	MLP	89.1	89.1	96.0	89.1	89.1	92.55
	KNN	84.7	83.5	94.1	83.6	83.5	88.8
	GB	85.6	85.3	94.4	85.3	85.3	89.85
	LR	86.5	86.2	94.8	86.3	86.2	90.5

Remarkable results were achieved by integrating different pre-trained models with Swin Transformers. The combination of MobileNetV2 with Swin and LR classifier achieved an impressive 98.5% ACC, while MobileNetV1 with Swin and LR achieved 98.4%. The performance of all CNN–ViT hybrid configurations across various classifiers is presented in Table 6, demonstrating the strong potential of these hybrid architectures for precise BC diagnosis. The proposed hybrid model significantly improved the accuracy and reliability of BC diagnosis from mammographic images by leveraging the strengths of each component.

Table 7 shows that highly approving outcomes were established. The integration of MobileNetV1, ViT, and a bagging LR classifier achieves a prominent overall ACC of 99%, surpassing all other tested architectures. This superior performance demonstrates the advantages of combining the local feature-extraction efficiency of MobileNetV1 with the global contextual representation learning of ViT, and the strong generalization capacity of bagging LR. This presentation balances precision and generalization by enabling the model to distinguish both fine structural features, such as variations in tissue texture and microcalcifications, and long-range relationships associated with the organization of breast tissue and lesion positioning.

The bagging ensemble further improves performance by averaging the predictions of many LR base methods. This reduces variation and enhances the model’s stability across folds. This ensemble technique is particularly effective for the imbalanced BI-RADS categories, as it reduces the probability of alignment toward more common classes while preserving SEN for high-risk, less normal classes. This study meticulously evaluated several combinations of pre-trained models, transformers, and ensemble classifiers, ultimately pinpointing a clear leader for clinical use. The MobileNetV1–ViT–Bagging LR configuration consistently outperformed the alternatives, indicating its potential as an effective and interpretable AI framework to assist radiologists in making early and precise BC diagnoses.

Table 8 summarizes the performance of the Bagging + LR model compared to SVM Linear for BI-RADS classification using deep features. The metrics are reported as the mean ± standard deviation across 5-fold cross-validation, with small standard deviations indicating stable, consistent performance. Bootstrap 95% confidence intervals were also computed, and the narrow intervals confirm the reliability and robustness of the model.

**Table 6:** The performance comparison of different pre-trained CNN models combined with the SWIN Transformer using various classifiers for BC classification.

Model	Classifier	PRE (%)	SEN (%)	SPE (%)	F1 (%)	ACC (%)	BACC (%)
EfficientNetB0+SWIN	SVM (Linear)	97.0	97.0	98.6	97.0	97.0	97.8
	SVM (Poly)	89.6	88.9	95.8	89.0	88.9	92.4
	SVM (RBF)	90.5	90.0	96.2	90.0	90.0	93.1
	DT	79.3	79.1	92.3	79.2	79.1	85.7
	RF	92.6	92.3	97.0	92.3	92.3	94.7
	MLP	96.9	96.9	98.8	96.9	96.9	97.9
	KNN	83.1	82.2	93.5	82.4	82.2	87.9
	GB	95.0	94.9	98.0	94.9	94.9	96.5
	LR	98.0	98.0	99.2	98.0	98.0	98.6
DenseNet121+SWIN	SVM (Linear)	97.2	97.2	98.8	97.2	97.2	98.0
	SVM (Poly)	92.7	92.4	97.1	92.4	92.4	94.8
	SVM (RBF)	93.1	92.9	97.3	92.8	92.9	95.1
	DT	78.6	78.3	92.0	78.3	78.3	85.2
	RF	92.3	92.0	96.9	92.0	92.0	94.5
	MLP	97.4	97.4	99.0	97.4	97.4	98.2
	KNN	86.9	85.8	95.0	85.8	85.8	90.4
	GB	94.7	94.7	98.0	94.7	94.7	96.4
	LR	98.3	98.3	99.3	98.3	98.3	98.8
InceptionV3+SWIN	SVM (Linear)	97.0	97.0	98.7	97.0	97.0	97.9
	SVM (Poly)	91.7	91.2	96.6	91.2	91.2	93.9
	SVM (RBF)	92.2	91.9	96.9	91.9	91.9	94.4
	DT	81.0	81.0	93.0	81.0	81.0	87.0
	RF	93.0	92.7	97.2	92.7	92.7	95.0
	MLP	97.1	97.1	98.9	97.1	97.1	98.0
	KNN	87.0	86.0	95.0	86.0	86.0	90.5
	GB	95.2	95.1	98.1	95.1	95.1	96.6
	LR	98.0	98.0	99.2	98.0	98.0	98.6
MobileNetV1+SWIN	SVM (Linear)	97.4	97.4	98.8	97.4	97.4	98.1
	SVM (Poly)	94.8	94.5	97.8	94.5	94.5	96.2
	SVM (RBF)	95.0	94.7	97.9	94.7	94.7	96.3
	DT	79.6	79.4	92.5	79.4	79.4	86.0
	RF	92.8	92.5	97.1	92.5	92.5	94.8
	MLP	97.5	97.5	99.1	97.5	97.5	98.3
	KNN	88.0	86.8	95.3	86.9	86.8	91.1
	GB	95.3	95.2	98.1	95.2	95.2	96.7
	LR	98.4	98.4	99.4	98.4	98.4	98.9

Model	Classifier	PRE (%)	SEN (%)	SPE (%)	F1 (%)	ACC (%)	BACC (%)
MobileNetV2+SWIN	SVM (Linear)	97.5	97.5	98.8	97.5	97.5	98.2
	SVM (Poly)	95.0	94.8	97.9	94.7	94.8	96.4
	SVM (RBF)	95.0	94.9	98.0	94.8	94.9	96.5
	DT	79.6	79.6	92.6	79.6	79.6	86.1
	RF	92.6	92.3	97.0	92.3	92.3	94.7
	MLP	97.0	97.0	98.9	97.0	97.0	98.0
	KNN	90.0	89.7	96.3	89.7	89.7	93.0
	GB	95.7	95.6	98.3	95.6	95.6	97.0
	LR	98.5	98.5	99.4	98.5	98.5	99.0
InceptionResNetV2+SWIN	SVM (Linear)	96.6	96.6	98.5	96.6	96.6	97.55
	SVM (Poly)	90.7	90.2	96.3	90.2	90.2	93.25
	SVM (RBF)	91.9	91.5	96.8	91.4	91.5	94.15
	DT	79.5	79.3	92.4	79.4	79.3	85.85
	RF	92.6	92.3	97.0	92.3	92.3	94.65
	MLP	96.2	96.2	98.6	96.2	96.2	97.40
	KNN	84.0	82.6	93.9	82.6	82.6	88.25
	GB	94.9	94.9	98.0	94.9	94.9	96.45
	LR	97.9	97.9	99.2	97.9	97.9	98.55
VGG16+SWIN	SVM (Linear)	97.3	97.3	98.9	97.3	97.3	98.10
	SVM (Poly)	88.3	87.6	95.2	87.7	87.6	91.40
	SVM (RBF)	90.9	90.6	96.5	90.6	90.6	93.55
	DT	79.5	79.5	92.6	79.5	79.5	86.05
	RF	92.3	92.0	96.9	92.0	92.0	94.45
	MLP	94.5	94.5	97.9	94.5	94.5	96.20
	KNN	83.9	82.4	93.7	82.5	82.4	88.05
	GB	95.2	95.1	98.1	95.1	95.1	96.60
	LR	98.1	98.1	99.2	98.1	98.1	98.65
VGG19+SWIN	SVM (Linear)	96.8	96.8	98.7	96.8	96.8	97.75
	SVM (Poly)	89.3	88.4	95.5	88.5	88.4	91.95
	SVM (RBF)	91.2	90.9	96.6	90.9	90.9	93.75
	DT	80.5	80.3	92.8	80.3	80.3	86.55
	RF	92.7	92.3	97.0	92.3	92.3	94.65
	MLP	94.5	94.5	98.0	94.5	94.5	96.25
	KNN	83.7	82.6	93.8	82.7	82.6	88.20
	GB	95.0	94.9	98.0	94.9	94.9	96.45
	LR	98.0	98.0	99.2	98.0	98.0	98.60

**Fig. 6:**
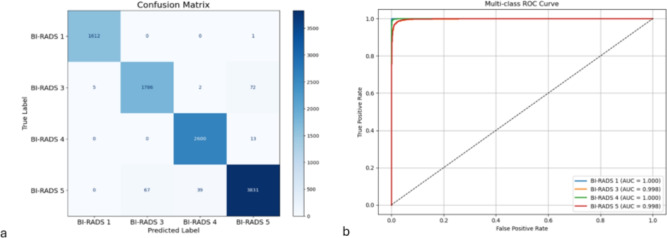
(**a**) The confusion matrix illustrating the multi-class BI-RADS classification performance of the proposed framework on the KAUBC dataset, highlighting correct predictions and limited misclassifications between clinically adjacent categories. (**b**) The class-wise ROC curves demonstrating the diagnostic discrimination capability of the proposed model across different BI-RADS categories.

Paired t-tests comparing Bagging LR with SVM Linear produced p-values exceeding 0.05 for all metrics, suggesting no statistically significant differences and underscoring the superior stability of Bagging LR. Furthermore, class-level AUC values are notably high (BI-RADS 1: 1.000, 3: 0.998, 4: 1.000, 5: 0.998), indicating exceptional discrimination across all categories, including rare and critical classes. The results indicate that the proposed model is robust, reproducible, and highly effective for multi-class BI-RADS classification. The proposed framework exhibits consistent performance across all BI-RADS classes, as summarized in Table 11 and illustrated in the confusion matrix (Fig. 6). This figure demonstrates the model’s ability to effectively differentiate among categories, with minimal misclassifications. This underscores the efficacy of the proposed feature fusion strategy and the Bagging LR classifier in addressing intra-class variability and alleviating class imbalance.

The results in Table 9 confirm the effectiveness of the hybrid framework within the KAUBC dataset, which combines Bagging with transformer-based models. The proposed structure effectively mitigates overfitting and addresses class imbalance. The configuration of MobileNetV1 with ViT and Bagging of LR yielded the highest ACC of 98.9% on this dataset among all tested integrations. The findings from the 5-fold cross-validation and the confusion matrix analysis validated the model’s stability under the assessed experimental conditions.

Table 10 presents the results of the ablation study assessing the role of each component in the proposed MobileNetV1–ViT framework. The results of single-backbone models demonstrate that ViT-only outperforms MobileNetV1-only across all evaluation metrics, highlighting the enhanced global representation ability of transformer-based features. Both individual models exhibit lower performance compared to the hybrid setups. The LR classifier implementation exhibits a notable performance drop, particularly with the MobileNetV1 backbone, underscoring the limited discriminative capacity of linear decision boundaries for complex mammographic features. The integration of PCA consistently enhances performance by reducing feature redundancy and improving generalization.

Each class achieved high values of ACC, SEN, SPE, and F1-score, confirming the model’s ability to differentiate between different BC risk levels. Notably, BI-RADS 4 (Suspicious Malignant) attained a recall of 98%, effectively reducing FNs in clinically high-risk cases. Furthermore, the Malignant and Likely Benign categories demonstrated near-optimal performance across the assessed metrics. Minor misclassifications predominantly occurred between clinically adjacent classes (e.g., Normal and Probably Benign), which exhibit overlapping imaging characteristics due to subtle visual differences, variations in tissue density, or borderline lesion appearance; nonetheless, no systematic or biased error patterns were detected.

The MobileNetV1 + ViT + PCA + Bagging framework was assessed through 5-fold cross-validation with various random seeds. Table 12 presents the performance metrics for each fold, encompassing ACCC, Precision, Recall, F1-Score, BACC, and the average SEN and SPE across all BI-RADS classes. The model demonstrates consistently high performance across all folds, with fold-wise accuracy ranging from 97.9% to 98.1% and an F1-Score of approximately 97.9%. The slight variations observed across folds reflect inherent differences in the training and test splits, suggesting that the model’s performance is stable and not dependent on any particular split.

The average ACC across folds is 98%, with a peak overall performance of 99% achieved through the optimal combination of random seed and fold. This indicates that the model achieves high accuracy in multi-class classification, while the marginally lower fold-wise averages provide a realistic evaluation of stability and generalization. The results demonstrate that the proposed framework achieves reliable, reproducible classification performance, with no evidence of overfitting or data leakage.

Table 13 compares the proposed hybrid approach with selected state-of-the-art models on the KAUBC dataset. The results demonstrate that our method achieves superior performance across multiple metrics, including accuracy, sensitivity, specificity, and XAI-enabled multi-class BI-RADS support.

**Table 7:** The comparison of performance metrics for various pre-trained CNN models combined with the ViT Transformer using different classifiers for breast cancer classification.

Model	Classifier	PRE (%)	SEN (%)	SPE (%)	F1 (%)	ACC (%)	BACC (%)
EfficientNetB0+ViT	SVM (Linear)	98.4	98.4	99.3	98.4	98.4	98.9
	SVM (Poly)	91.1	90.5	96.3	90.6	90.5	93.4
	SVM (RBF)	93.1	92.8	97.2	92.7	92.8	95.0
	DT	88.9	88.9	96.0	88.9	88.9	92.5
	RF	96.3	96.2	98.5	96.1	96.2	97.4
	MLP	98.1	98.1	99.3	98.1	98.1	98.7
	KNN	83.9	82.9	93.8	83.0	82.9	88.4
	GB	97.8	97.8	99.1	97.8	97.8	98.5
	LR	98.4	98.4	99.4	98.4	98.4	98.9
	Bagging	98.4	98.4	99.3	98.4	98.4	98.9
DenseNet121+ViT	SVM (Linear)	98.0	98.0	99.1	98.0	98.0	98.6
	SVM (Poly)	93.5	93.2	97.4	93.2	93.2	95.3
	SVM (RBF)	93.8	93.6	97.5	93.5	93.6	95.6
	DT	88.6	88.5	95.8	88.5	88.5	92.2
	RF	96.6	96.4	98.6	96.4	96.4	97.5
	MLP	98.1	98.1	99.3	98.1	98.1	98.7
	KNN	87.3	86.1	95.1	86.2	86.1	90.6
	GB	97.4	97.4	99.0	97.4	97.4	98.2
	LR	98.2	98.2	99.3	98.2	98.2	98.8
	Bagging	97.9	97.9	99.1	97.9	97.9	98.5
InceptionV3+ViT	SVM (Linear)	97.8	97.8	99.2	97.8	97.8	98.5
	SVM (Poly)	93.4	93.0	97.3	93.0	93.0	95.2
	SVM (RBF)	94.7	94.5	97.9	94.4	94.5	96.2
	DT	88.5	88.4	95.8	88.4	88.4	92.1
	RF	96.4	96.2	98.5	96.1	96.2	97.4
	MLP	98.1	98.1	99.3	98.1	98.1	98.7
	KNN	87.6	86.5	95.2	86.5	86.5	90.9
	GB	97.4	97.4	99.0	97.4	97.4	98.2
	LR	98.1	98.1	99.3	98.1	98.1	98.7
	Bagging	98.2	98.2	99.3	98.2	98.2	98.8
MobileNetV1+ViT	SVM (Linear)	98.4	98.4	99.3	98.4	98.4	98.9
	SVM (Poly)	95.1	94.8	97.9	94.7	94.8	96.4
	SVM (RBF)	95.4	95.1	98.0	95.0	95.1	96.6
	DT	89.0	88.9	96.0	88.9	88.9	92.5
	RF	96.6	96.4	98.6	96.4	96.4	97.5
	MLP	98.3	98.3	99.3	98.3	98.3	98.8
	KNN	88.2	87.0	95.4	87.1	87.0	91.2
	GB	97.5	97.5	99.0	97.5	97.5	98.3
	LR	98.9	98.9	99.6	98.9	98.9	99.3
	**Bagging**	**99.0**	**99.0**	**99.5**	**99.0**	**99.0**	**99.3**

Model	Classifier	PRE (%)	SEN (%)	SPE (%)	F1 (%)	ACC (%)	BACC (%)
MobileNetV2+ViT	SVM (Linear)	98.2	98.2	99.3	98.2	98.2	98.8
	SVM (Poly)	95.3	95.1	98.1	95.1	95.1	96.6
	SVM (RBF)	95.5	95.4	98.2	95.4	95.4	96.8
	DT	89.7	89.6	96.2	89.6	89.6	92.9
	RF	96.4	96.2	98.5	96.2	96.2	97.4
	MLP	98.1	98.1	99.3	98.1	98.1	98.7
	KNN	90.6	90.0	96.4	90.0	90.0	93.2
	GB	97.6	97.6	99.0	97.6	97.6	98.3
	LR	98.3	98.3	99.4	98.3	98.3	98.9
	Bagging	98.3	98.3	99.3	98.3	98.3	98.8
InceptionResNetV2+ViT	SVM (Linear)	98.1	98.1	99.2	98.1	98.1	98.7
	SVM (Poly)	92.8	92.4	97.0	92.3	92.4	94.7
	SVM (RBF)	93.5	93.2	97.4	93.2	93.2	95.3
	DT	89.3	89.2	96.1	89.3	89.3	92.7
	RF	96.4	96.2	98.5	96.2	96.2	97.4
	MLP	97.9	97.9	99.2	97.9	97.9	98.6
	KNN	85.1	83.5	94.2	83.5	83.5	88.9
	GB	97.5	97.5	99.0	97.5	97.5	98.3
	LR	98.4	98.4	99.4	98.4	98.4	98.9
	Bagging	98.2	98.2	99.2	98.2	98.2	98.7
VGG16+ViT	SVM (Linear)	98.4	98.4	99.3	98.4	98.4	98.9
	SVM (Poly)	88.4	87.7	95.3	87.7	87.7	91.5
	SVM (RBF)	91.4	91.1	96.6	91.1	91.1	93.9
	DT	90.2	90.2	96.4	90.2	90.2	93.3
	RF	96.3	96.1	98.4	96.0	96.1	97.3
	MLP	96.4	96.4	98.7	96.4	96.4	97.6
	KNN	83.9	82.4	93.7	82.5	82.4	88.1
	GB	97.4	97.4	99.0	97.4	97.4	98.2
	LR	98.7	98.7	99.5	98.7	98.7	99.1
	Bagging	98.7	98.7	99.3	98.7	98.7	99.0
VGG19+ViT	SVM (Linear)	98.4	98.4	99.4	98.4	98.4	98.9
	SVM (Poly)	89.4	88.6	95.6	88.7	88.6	92.1
	SVM (RBF)	91.9	91.5	96.8	91.5	91.5	94.2
	DT	90.1	90.0	96.4	90.0	90.0	93.2
	RF	96.7	96.5	98.6	96.5	96.5	97.6
	MLP	95.7	95.7	98.4	95.7	95.7	97.1
	KNN	83.8	82.6	93.8	82.7	82.6	88.2
	GB	97.3	97.3	99.0	97.3	97.3	98.2
	LR	98.1	98.1	99.3	98.1	98.1	98.7
	Bagging	98.4	98.4	99.3	98.4	98.4	98.9

**Table 8:** The comprehensive evaluation of the proposed Bagging + LR model for BI-RADS classification using deep features. Performance metrics are reported as mean ± standard deviation across 5-fold cross-validation, along with 95% bootstrap confidence intervals. Paired t-tests were conducted against the baseline SVM (Linear), and class-level AUC values are also reported.

Metric	Bagging + LR	Bootstrap 95% CI	SVM Baseline	p-value	AUC per class
ACC	0.9802 ± 0.0018	0.9782 – 0.9837	0.9808 ± 0.0037	0.6805	–
Precision	0.9801 ± 0.0018	0.9784 – 0.9836	0.9808 ± 0.0037	0.6495	–
Recall	0.9802 ± 0.0018	0.9781 – 0.9836	0.9808 ± 0.0037	0.6805	–
F1-score	0.9801 ± 0.0018	0.9783 – 0.9836	0.9807 ± 0.0037	0.6666	–
SPE	0.9914 ± 0.0009	0.9904 – 0.9930	0.9917 ± 0.0019	0.6749	–
AUC (per class)	–	–	–	–	BI-RADS 1: 1.000, 3: 0.998, 4: 1.000, 5: 0.998

**Table 9:** The hyperparameter settings for the ViT Transformer and MobileNetV1 models used in the breast cancer classification experiments. Data augmentation included rescaling, rotation, flipping, and intensity adjustments. Early stopping was applied with a patience of 5 based on the validation loss.

Hyperparameter	ViT Transformer	MobileNetV1
Learning Rate	0.00001	0.001
Optimizer	AdamW	SGD
Weight Decay	0.01	0.001
Dropout Rate	0.1	0.5
Batch Size	32	32
Epochs	50 (with early stopping)	50 (with early stopping)
Loss Function	Categorical Cross-Entropy	Categorical Cross-Entropy
Patch Size	4 $\times$ 4	—
Window Size	7 $\times$ 7	—

**Table 10:** The ablation study evaluating the contribution of different components in the proposed MobileNetV1–ViT framework. Results are reported as mean ± standard deviation across five-fold cross-validation.

Model Variant	ACC (%)	SEN (%)	SPE (%)	Precision	F1-score	BACC (%)
MobileNetV1 only	97	97.1	99	98	98	98.5
ViT only	98.5	98.8	99.2	99	99	99
MobileNetV1 + LR	92.7	92.7	97.2	92.9	92.7	95
ViT + LR	98.7	98	99	98.8	98.9	98.5
MobileNetV1 + PCA + LR	94	93.9	98	94	94	94
MobileNetV1 + ViT + PCA + LR	98.3	98.3	99.4	98.3	98.3	98.8
MobileNetV1 + ViT + PCA + SVM	98.1	98.1	99.3	98.2	98.2	98.6
**MobileNetV1 + ViT + PCA + Bagging (Ours)**	**99**	**99**	**99.5**	**99**	**99**	**99.3**

**Table 11:** The per-class performance metrics for the classification model.

Class	PRE (%)	SEN (%)	SPE (%)	F1-Score (%)	ACC (%)	BACC (%)
Malignant	100	100	100	100	100	100
Normal	98	98	100	98	98	99
Probably Benign	99	100	100	100	100	100
Suspicious Malignant	99	98	99	98	98	98.5

**Table 12:** The evaluation metrics of the proposed model across 5-fold cross-validation. Average SEN and SPE are calculated across all BI-RADS classes.

Fold	ACC (%)	Precision (%)	Recall (%)	F1-Score (%)	Balanced Acc (%)	SEN (%)	Avg SPE (%)
Fold-1	97.91	97.91	97.91	97.90	98.69	98.35	99.05
Fold-2	97.86	97.85	97.86	97.85	98.57	98.20	98.95
Fold-3	98.35	98.36	98.35	98.35	98.98	98.50	99.10
Fold-4	98.00	97.99	98.00	98.00	98.66	98.35	99.07
Fold-5	97.96	97.95	97.96	97.95	98.63	98.40	99.00
**Average**	98.02	98.03	98.02	98.03	98.71	98.36	99.03

**Table 13:** The comparative evaluation of the proposed hybrid approach against selected state-of-the-art models on the KAUBC dataset.

Study	Model/architecture	Dataset used	Key performance metrics
Eskandari et al. ^58^	MobileNetV1 + ViT Transformer	IDC histopathological images	Validation ACC: 93%.
Alotaibi et al. ^22^	ViT + DeiT (Ensemble by Averaging)	BreakHis dataset	ACC: 98.17%; F1-score: 98.12%.
Ahmed et al. ^19^	MAX-ViT + GAFM + HHO + XGBoost	KAUBC mammograms	ACC: 98.2%; Precision: 98.0%; Recall: 98.1%; F1-score: 98.0%; AUC: 98.9%; MCC: 95%.
Wani et al. (2024)^33^	Hybrid CNN + LightGBM with SHAP explanations	Real-world BC dataset	ACC: 98.3%; Precision: 98.7%; SEN: 98.7%; F1: 0.987; Binary BC classification, XAI-enabled.
**Proposed Method**	**MobileNetV1 + ViT Transformer + Bagging (LR)**	**KAUBC mammograms**	**ACC: 99.0%; SEN: 99.0%; SPE: 99.5%; Multi-class BI-RADS support; XAI-enabled.**

### Analysis of misclassified cases

**Fig. 7:**
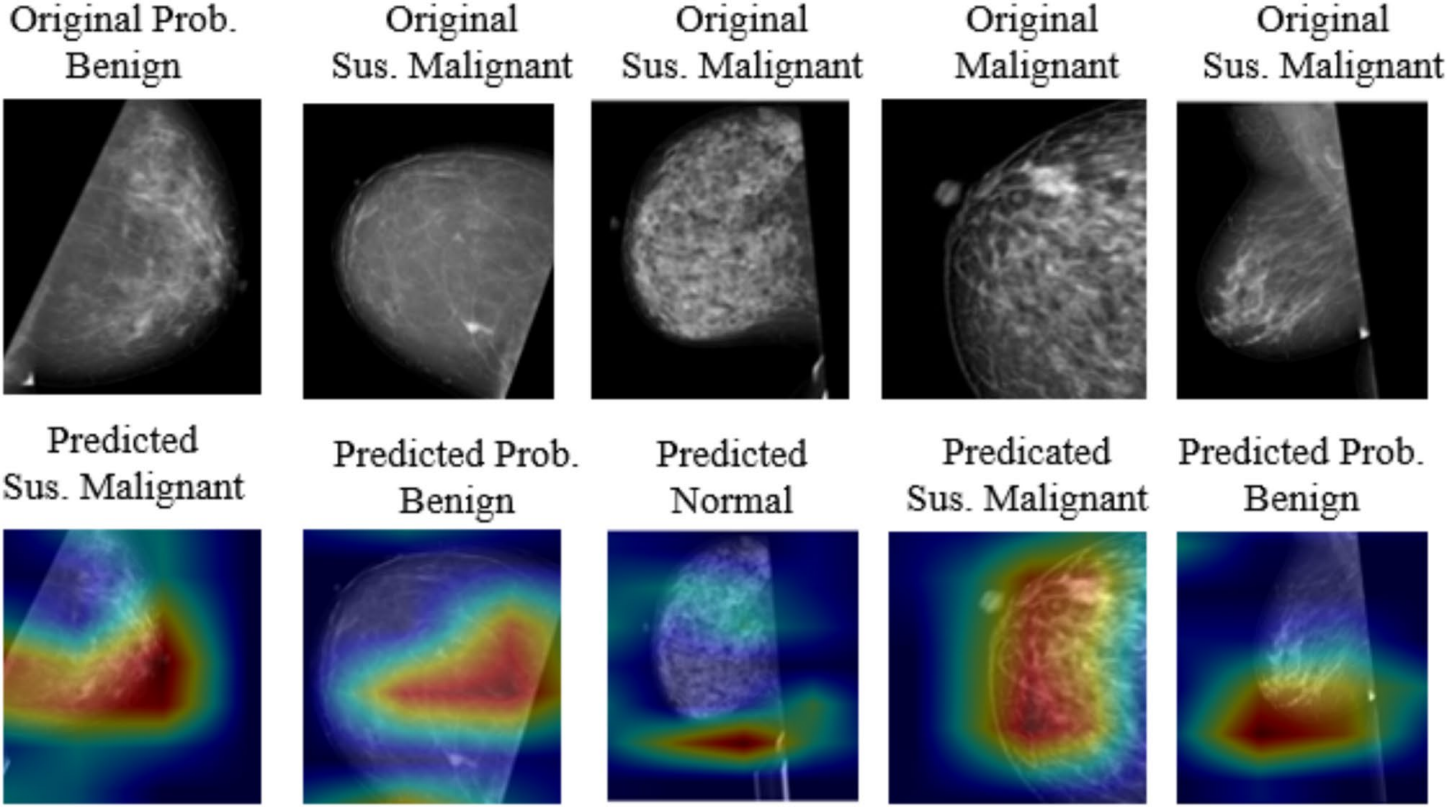
The representative XAI visualizations for misclassified mammogram cases.

We conducted an analysis of representative misclassified samples generated by the Bagging + LR classifier to gain a deeper understanding of the proposed framework’s behavior. Table 14 and Fig. 7 demonstrate that the majority of misclassifications arise in visually ambiguous scenarios, characterized by dense tissue or low-contrast lesions, which result in partial activation discrepancies in the Grad-CAM maps. Misclassifications predominantly occurred between adjacent BI-RADS categories, particularly between Normal, Probably Benign, and Suspicious Malignant, highlighting the challenges of differentiating subtle or borderline mammographic findings. These errors are frequently linked to dense breast tissue, low-contrast or small lesions, and subtle calcifications, which pose detection challenges even in clinical practice.

This error analysis demonstrates that the identified misclassifications are clinically plausible and align with established diagnostic challenges in mammography interpretation. Borderline cases underscore situations in which additional clinical context or expert evaluation may be necessary, underscoring the need for careful interpretation in automated BC diagnosis systems.

**Table 14:** The misclassified mammogram cases corresponding to the Grad-CAM visualizations shown in Fig. 7.

Index	True label	Predicted label	Observation
777	Probably Benign	Suspicious Malignant	Dense fibroglandular tissue mimicking suspicious patterns
133	Suspicious Malignant	Probably Benign	Lesion boundary partially obscured by surrounding tissue
161	Suspicious Malignant	Normal	Low-contrast lesion with weak visual saliency
806	Malignant	Suspicious Malignant	Diffuse mass appearance causing uncertainty in risk level
43	Suspicious Malignant	Probably Benign	Attention spread over dense tissue rather than lesion core

### Explainability and ethical considerations using Grad-CAM and Grad-CAM++

Grad-CAM and Grad-CAM++ visualization tools were utilized to demonstrate the application of the proposed hybrid DL model and to elucidate its decision-making process. The techniques generate class-discriminative heatmaps that highlight regions in mammography images that significantly influence the model’s classification results^27^. Visual explanations enable radiologists to correlate the model’s focus areas with their clinical knowledge, thereby improving trust and interpretability in AI-assisted diagnosis.

The examination of Grad-CAM and Grad-CAM++ heatmaps across different BI-RADS categories demonstrated the model’s ability to highlight clinically relevant features. In patients with malignant conditions, both methods reveal significant activation in areas with rare masses and clustered calcifications, aligning with radiological annotations of malignancy. This alignment demonstrates that the model’s predictions are based on medically relevant patterns rather than extraneous visual artifacts. In contrast, the benign class heatmaps demonstrated significant activation in dense parenchymal areas, without focusing on isolated mass-like features. This is consistent with radiologists’ views on benign conditions.

Grad-CAM and Grad-CAM++ showed low activation across the entire breast region in normal images, which was appropriate given the lack of abnormal tissue patterns. In the comparison of the two methods, Grad-CAM demonstrated enhanced localization of suspicious areas, effectively balancing computational efficiency and diagnostic relevance. Grad-CAM++ enhanced localization and increased SEN at subtle lesion boundaries, effectively demonstrating that the proposed model focuses on clinically significant areas, such as masses, calcifications, and tissue asymmetries, thereby offering visual interpretability and improving relevant indicators.

**Fig. 8:**
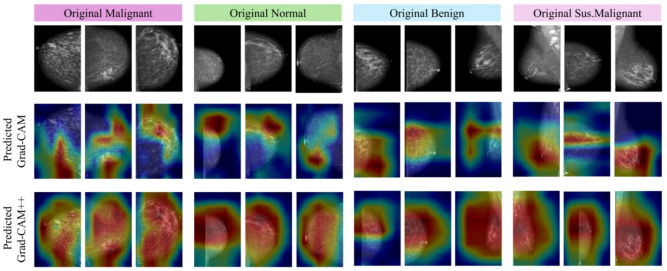
The representative Grad-CAM and Grad-CAM++ heatmaps for malignant, suspicious malignant, benign, and normal mammogram images. Warmer colors (red, orange, and yellow) indicate stronger model attention and higher diagnostic relevance, while cooler colors (green and blue) indicate lower activation.

The model’s visual interpretability increases confidence in its predictions and supports its incorporation into clinical workflows, where explainable artificial intelligence is crucial for diagnostic validation. Figure 8 illustrates the Grad-CAM and Grad-CAM++ heatmaps corresponding to the four BI-RADS classes. This figure illustrates the proposed model’s emphasis on clinically significant areas, including masses, calcifications, and tissue asymmetries, thereby enhancing visual interpretability and reinforcing the reliability of model predictions within a diagnostic framework.

Ethical considerations are essential in the application of medical AI. The incorporation of XAI techniques, including Grad-CAM and Grad-CAM++, enhances trust by visually highlighting the regions that affect model predictions, enabling clinicians to confirm that the model targets pertinent mammographic features. In this study, a seasoned radiologist evaluated the model outputs to validate the clinical significance of the highlighted regions. Ethical concerns, including data privacy, annotation transparency, and the responsible use of AI in clinical decision-making, are examined. The KAUBC dataset has been fully anonymized, and all experiments adhere to institutional ethical standards. XAI plays a crucial role in enhancing interpretability, accountability, and the safe deployment of AI-based BC screening tools.

## Discussion

This section evaluates the performance of several pre-trained DL models, classifiers, and their fusion for BC classification on mammographic images within the proposed DL framework. A series of assessments was conducted to compare various feature-extraction architectures, including Inception, MobileNet, VGG, InceptionResNetV2, DenseNet, EfficientNet, ViT Transformers, and Swin Transformers.

We employed various ML classifiers on these models to evaluate their effectiveness in distinguishing among distinct BI-RADS categories in mammography scans. The results presented in Table 4 provide valuable insights into each model’s effectiveness at identifying discriminative features. The subsequent transformer-augmented configurations represent the most dependable diagnostic setups. All pre-trained CNNs exhibited a trade-off between computational efficiency and diagnostic ACC. Deep architectures, such as VGG, effectively capture complicated texture patterns; however, they require substantial computational resources. Lightweight variants, such as MobileNet, achieve an optimal balance between precision and efficiency, making them suitable for real-time clinical applications. The proposed system selected MobileNet as the primary feature extractor due to its favorable trade-off between high diagnostic accuracy and low computational cost. While it may not identify as many subtle patterns as more complex networks, its speed and ACC render it a suitable option for practical medical imaging applications.

The connection between classifiers and the features extracted influenced the overall validation of the diagnosis. Table 5 illustrates that specific fusion, especially MobileNetV1 with SVM, gave strong results on the KAUBC dataset, highlighting the relevance of carefully selecting classifier–model connections. In our experiments, SVMs outperformed alternative classifiers when integrated with transformer-enhanced features, likely due to their ability to handle high-dimensional feature spaces and to generate effective decision boundaries within the evaluated dataset.

The incorporation of Vision and Swin Transformers significantly improved categorization ACC by leveraging their ability to capture long-range dependencies and global contextual information, complementing the strengths of CNNs in obtaining localized spatial details. Tables 6 and 7 indicate that Swin Transformers outperformed traditional ViT Transformers. The sequence design likely enables the learning of features at varying scales, thereby facilitating more accurate spatial performance. The proposed dual-stream architecture, combining MobileNetV1 with ViT, leverages the strengths of both CNNs and transformers to enhance mammogram interpretation.

In this configuration, MobileNetV1 effectively identified small local features critical for detecting microcalcifications and focal abnormalities. ViT effectively captured a broader global context, enhancing our understanding of tissue structures. This hybrid integration enabled the precise identification of both minor lesions and significant issues, thereby enhancing overall diagnostic ACC. ViT Transformers typically require large datasets to generalize effectively because they lack categorical alignment. However, integrating structural priors from CNNs reduces this issue. The MobileNetV1–ViT hybrid achieved superior efficiency in distinguishing benign from malignant instances, thereby enhancing both diagnostic interpretation and ACC. The hybrid MobileNetV1–ViT architecture achieved significant performance gains when combined with a bagging classifier.

Bagging improved model performance by aggregating predictions from multiple base learners, reducing overfitting, and yielding more stable results on the KAUBC dataset. This ensemble technique enhanced feature diversity and increased model robustness, achieving high ACC across malignant, normal, probably benign, and suspicious malignant categories in the evaluated dataset, as shown in Table 5.

The proposed framework focuses on image-level BI-RADS classification rather than explicit lesion detection; however, the hybrid CNN–Transformer architecture enables the model to attend to multiple spatially distinct regions within a single mammogram. Grad-CAM and Grad-CAM++ visualizations demonstrate that, in cases of heterogeneous or multifocal abnormalities, the model can simultaneously focus on multiple suspicious regions. This indicates a strong capability in managing mammograms that may include multiple small tumors, although the current study does not address lesion-level localization and enumeration.

This study emphasized the significance of explainability. We employed Grad-CAM and Grad-CAM++ visualizations to elucidate the model’s decision-making process on the KAUBC dataset. The heatmaps generated by this methodology indicate that the model primarily focuses on regions corresponding to irregular masses and clustered calcifications within this dataset. Grad-CAM offered a broad yet characteristic set of features, whereas Grad-CAM++ produced more detailed and precise attention maps, facilitating the identification of lesion boundaries and subtle changes in the evaluated cases. The results suggest that the proposed approach demonstrates interpretability aligned with the diagnostic patterns observed by experienced radiologists in this dataset.

The KAUBC dataset offers annotations solely in BI-RADS categories, lacking histopathological or molecular BC subtype labels such as Luminal A/B, HER2-positive, or triple-negative. Thus, the performance evaluation in this study focuses on radiological risk assessment rather than subtype-specific diagnosis, an area of significant future research that could leverage datasets with more comprehensive clinical annotations.

Although we report ACC, SEN, SPE, and F1-score to evaluate model performance, we acknowledge that additional metrics, such as Matthews correlation coefficient (MCC) and AUC, could offer further insights, particularly in the context of imbalanced datasets. These metrics can complement traditional measures and provide a more comprehensive assessment of model effectiveness; their inclusion is therefore suggested for future studies.

The lightweight design of MobileNetV1, in conjunction with transformer-based feature fusion, facilitates efficient inference from a clinical deployment perspective. The proposed framework enables rapid image processing, facilitating the analysis of a complete four-view mammographic examination (CC and MLO for both breasts) in less than 1 second on standard GPU hardware. This throughput level facilitates the potential incorporation of the system into standard clinical decision-support workflows.

The reported performance is based on cross-validation results from a clinically curated BI-RADS dataset and does not necessarily generalize to all real-world screening populations without additional external validation. Although the results are promising, implementing the MobileNetV1–ViT–Bagging system in medical contexts poses numerous challenges. Ensuring ethical and safe outcomes requires protecting data privacy and complying with healthcare regulations, including HIPAA, as well as applicable local governance frameworks.

Differences in mammography equipment, imaging protocols, and patient demographics across medical facilities can affect image quality and the model’s effectiveness. To maintain flexibility and generalization across diverse contexts, continuous retraining and scaling on diverse datasets, including KAUBC, are essential. Trust and acceptance from physicians are essential components for successful therapeutic integration. Radiologists will have access to feature contributions and attention maps via XAI to obtain transparent, comprehensible model results. This will enhance their confidence and facilitate collaboration with patients in decision-making. Ethical and operational factors must also inform the incorporation of AI-driven solutions in healthcare. The model should enhance, rather than supplant, radiologists’ skills, ensuring human oversight and accountability in clinical decision-making. Mitigating algorithmic bias is crucial to ensuring equitable diagnostic performance across diverse populations and imaging scenarios, thereby reducing inequities in clinical outcomes.

The suggested MobileNetV1 + ViT + PCA + Bagging framework exhibits strong and superior performance in multi-class BI-RADS classification. In 5-fold cross-validation, the fold-wise accuracy ranged from 97.9% to 98.1%, while the F1-Scores remained consistently around 97.9%. The small discrepancies among folds indicate inherent variability in the data partitions, affirming the model’s robustness. The fold-average ACC is 98%, although the model achieved a maximum of 99% for certain seeds and folds, underscoring its ability to achieve precise classification. These results demonstrate consistent, replicable performance, with no indications of overfitting or data leakage.

## Conclusion

This study presents an efficient hybrid DL framework for BC diagnosis using mammographic images. The proposed architecture integrates MobileNetV1 for efficient local feature extraction with a ViT to capture global contextual representations, while a bagging ensemble classifier further enhances classification reliability. Experimental evaluation on the KAUBC dataset demonstrates strong diagnostic performance, with ACC, SEN, and SPE of 99%, 99%, and 99.5%, respectively. These results highlight the effectiveness of combining convolutional and transformer-based models for robust BI-RADS classification. The dual-stream design leverages complementary feature representations, reducing FB and improving robustness in challenging scenarios such as dense breast tissue and early-stage abnormalities. Despite promising performance, several limitations should be acknowledged. First, the lack of external clinical validation is a key constraint, as all experiments were conducted solely on the KAUBC dataset. Although this dataset is clinically annotated using BI-RADS categories, it primarily comprises patients from the Jeddah region of Saudi Arabia, limiting geographic and racial diversity and potentially affecting the generalizability of the proposed framework. In addition, class imbalance across BI-RADS categories, particularly the underrepresentation of BI-RADS 4 and 5 cases, may compromise classification robustness for minority high-risk classes, despite the use of data augmentation, class weighting, cross-validation, and early stopping. Furthermore, the proposed framework is designed for image-level BI-RADS classification and does not perform explicit lesion-level detection or localization. Consequently, precise localization or enumeration of multifocal abnormalities within a single mammogram remains beyond the scope of the current study. Although Grad-CAM and Grad-CAM++ visualizations indicate that the model attends to diagnostically relevant regions, explicit lesion-level supervision constitutes an important direction for future investigation. Moreover, the KAUBC dataset lacks histopathological or molecular annotations of BC subtypes (e.g., Luminal A/B, HER2-positive, or triple-negative), limiting evaluation to radiological measures and preventing subtype-specific diagnostic analysis. In addition, the adopted preprocessing steps, including image resizing and grayscale conversion, may introduce a partial loss of dynamic range and attenuate very subtle diagnostic features in mammographic images. Although mammograms are inherently acquired in grayscale, and contrast enhancement techniques were applied to mitigate these effects, slight degradation of fine textural details cannot be entirely excluded. This represents a recognized limitation of the current preprocessing pipeline. The current study is confined to mammography images and does not incorporate complementary imaging modalities such as US, MRI, or DBT, which could provide additional diagnostic insights in clinical practice. From a computational perspective, certain components of the hybrid architecture, particularly the Vision Transformer, increase computational complexity and may pose deployment challenges in resource-limited clinical environments. Additionally, the framework assumes the presence of high-quality DICOM images; degraded or noisy acquisitions may adversely affect classification performance. Although multiple evaluation metrics were reported, incorporating supplementary measures, such as MCC and class-wise AUC, could further refine the interpretation of performance on highly imbalanced datasets. Future research will focus on validating the proposed framework using independent, multi-center datasets and conducting prospective clinical studies in collaboration with radiologists to assess real-world applicability. Additional directions include lesion-level detection and localization, subtype-aware learning using datasets with pathological annotations, multi-modal fusion of mammography with ultrasound or MRI, and systematic robustness evaluation under variations in image contrast, brightness, and resolution. Ultimately, efforts will focus on optimizing model complexity to enhance computational efficiency, scalability, and clinical throughput, thereby facilitating the integration of reliable, high-performance hybrid DL models into routine BC screening workflows.

## Supplementary Information

Supplementary Information.

## Data Availability

All results for the present study are included in this published version of the article. The analyzed dataset is freely available at https://www.kaggle.com/datasets/asmaasaad/king-abdulaziz-university-mammogram-dataset and can be requested on reasonable correspondence.

## References

[1] Sung, H. et al. Global cancer statistics 2020: Globocan estimates of incidence and mortality worldwide for 36 cancers in 185 countries. *CA Cancer J. Clin.* **71**, 209–249 (2021). 33538338 10.3322/caac.21660

[2] Chandy, A. et al. A review on iot based medical imaging technology for healthcare applications. *J. Innov. Image Process. (JIIP)* **1**, 51–60 (2019).

[3] D’Orsi, C. J., Bassett, L. & Feig, S. A. *ACR BI-RADS Atlas: Breast Imaging Reporting and Data System; Mammography, Ultrasound, Magnetic Resonance Imaging, Follow-up and Outcome Monitoring, Data Dictionary* (American College of Radiology, 2013).

[4] Chen, C. et al. Deep learning prediction of mammographic breast density using screening data. *Sci. Rep.* **15**, 11602 (2025). 40185813 10.1038/s41598-025-95275-5 PMC11971370

[5] Ren, T., Gao, Z., Yang, L., Cheng, W. & Luo, X. Development of a nomogram for predicting malignancy in bi-rads 4 breast lesions using contrast-enhanced ultrasound and shear wave elastography parameters. *Sci. Rep.* **15**, 1356 (2025). 39779822 10.1038/s41598-025-85862-x PMC11711183

[6] van den Kroonenberg, D. L. et al. Mp30-02 retrospective validation of a computer aided diagnosis system based on multiparametric transrectal ultrasound for the localization of clinically significant prostate cancer. *J. Urol.* **211**, e490 (2024).

[7] Porkar, P. et al. Enhancing cancer zone diagnosis in mri images: A novel som neural network approach with block processing in the presence of noise. *Iran. J. Blood Cancer* **17**, 34–45 (2025).

[8] Rafie, Z. et al. Leveraging xgboost and explainable ai for accurate prediction of type 2 diabetes. *BMC Public Health* **25**, 3688 (2025). 41174635 10.1186/s12889-025-24953-w PMC12577272

[9] Chen, L. Medical education and artificial intelligence: Question answering for medical questions based on intelligent interaction. *Concurren. Comput. Pract. Exp.* **36**, e8079 (2024).

[10] Litjens, G. et al. A survey on deep learning in medical image analysis. *Med. Image Anal.* **42**, 60–88 (2017). 28778026 10.1016/j.media.2017.07.005

[11] Elmore, J. G. et al. Diagnostic concordance among pathologists interpreting breast biopsy specimens. *JAMA* **313**, 1122–1132 (2015). 25781441 10.1001/jama.2015.1405 PMC4516388

[12] Becker, A. S. et al. Deep learning in mammography: Diagnostic accuracy of a multipurpose image analysis software in the detection of breast cancer. *Invest. Radiol.* **52**, 434–440 (2017). 28212138 10.1097/RLI.0000000000000358

[13] Rodriguez-Ruiz, A. et al. Stand-alone artificial intelligence for breast cancer detection in mammography: Comparison with 101 radiologists. *JNCI J. Natl. Cancer Inst.* **111**, 916–922 (2019). 30834436 10.1093/jnci/djy222 PMC6748773

[14] Islam, T. et al. Predictive modeling for breast cancer classification in the context of Bangladeshi patients by use of machine learning approach with explainable ai. *Sci. Rep.* **14**, 8487 (2024). 38605059 10.1038/s41598-024-57740-5 PMC11009331

[15] Chang, Y.-W. et al. Artificial intelligence for breast cancer screening in mammography (ai-stream): Preliminary analysis of a prospective multicenter cohort study. *Nat. Commun.* **16**, 2248 (2025). 40050619 10.1038/s41467-025-57469-3 PMC11885569

[16] Chai, J., Zeng, H., Li, A. & Ngai, E. W. Deep learning in computer vision: A critical review of emerging techniques and application scenarios. *Mach. Learn. Appl.* **6**, 100134 (2021).

[17] Arık, O. A., Schutten, M. & Topan, E. Weighted earliness/tardiness parallel machine scheduling problem with a common due date. *Expert Syst. Appl.* **187**, 115916 (2022).

[18] Shorten, C. & Khoshgoftaar, T. M. A survey on image data augmentation for deep learning. *J. Big Data* **6**, 1–48 (2019). 10.1186/s40537-021-00492-0 PMC8287113 34306963

[19] Ahmed, S., Elazab, N., El-Gayar, M. M., Elmogy, M. & Fouda, Y. M. Multi-scale vision transformer with optimized feature fusion for mammographic breast cancer classification. *Diagnostics* **15**, 1361 (2025). 40506933 10.3390/diagnostics15111361 PMC12155438

[20] Abimouloud, M. L., Bensid, K., Elleuch, M., Aiadi, O. & Kherallah, M. Vision transformer-convolution for breast cancer classification using mammography images: A comparative study. *Int. J. Hybrid Intell. Syst.* **20**, 67–83 (2024).

[21] Voon, W. et al. Performance analysis of seven convolutional neural networks (cnns) with transfer learning for invasive ductal carcinoma (idc) grading in breast histopathological images. *Sci. Rep.* **12**, 19200 (2022). 36357456 10.1038/s41598-022-21848-3 PMC9649772

[22] Alotaibi, A. *et al.* Vit-deit: An ensemble model for breast cancer histopathological images classification. In *2023 1st International Conference on Advanced Innovations in Smart Cities (ICAISC)* 1–6 (IEEE, 2023).

[23] Nguyen, H. T., Tran, S. B., Nguyen, D. B., Pham, H. H. & Nguyen, H. Q. A novel multi-view deep learning approach for bi-rads and density assessment of mammograms. In *2022 44th Annual International Conference of the IEEE Engineering in Medicine & Biology Society (EMBC)* 2144–2148 (IEEE, 2022). 10.1109/EMBC48229.2022.9871564 36085843

[24] Diwakaran, M. & Surendran, D. Breast cancer prognosis based on transfer learning techniques in deep neural networks. *Inf. Technol. Control* **52**, 381–396 (2023).

[25] Qasrawi, R. et al. Hybrid ensemble deep learning model for advancing breast cancer detection and classification in clinical applications. *Heliyon* **10**, e38374 (2024). 39398009 10.1016/j.heliyon.2024.e38374 PMC11467543

[26] Al-Thelaya, K. et al. Applications of discriminative and deep learning feature extraction methods for whole slide image analysis: A survey. *J. Pathol. Inform.* **14**, 100335 (2023). 37928897 10.1016/j.jpi.2023.100335 PMC10622844

[27] Hasan, M. M. et al. Fp-cnn: Fuzzy pooling-based convolutional neural network for lung ultrasound image classification with explainable ai. *Comput. Biol. Med.* **165**, 107407 (2023). 37678140 10.1016/j.compbiomed.2023.107407

[28] Lee, Z. Y., Goh, Y. L. E. & Lai, C. Classification of mammographic breast density and its correlation with bi-rads in elder women using machine learning approach. *J. Med. Imaging Radiat. Sci.* **53**, 28–34 (2022). 34801440 10.1016/j.jmir.2021.10.004

[29] Hamyoon, H. et al. Artificial intelligence, bi-rads evaluation and morphometry: A novel combination to diagnose breast cancer using ultrasonography, results from multi-center cohorts. *Eur. J. Radiol.* **157**, 110591 (2022). 36356463 10.1016/j.ejrad.2022.110591

[30] Tsai, K.-J. et al. A high-performance deep neural network model for bi-rads classification of screening mammography. *Sensors* **22**, 1160 (2022). 35161903 10.3390/s22031160 PMC8838754

[31] Carrilero-Mardones, M., Parras-Jurado, M., Nogales, A., Pérez-Martín, J. & Díez, F. J. Deep learning for describing breast ultrasound images with bi-rads terms. *J. Imaging Inform. Med.* **37**, 2940–2954 (2024). 38926264 10.1007/s10278-024-01155-1 PMC11612129

[32] Shi, J. et al. Quaternion grassmann average network for learning representation of histopathological image. *Pattern Recogn.* **89**, 67–76 (2019).

[33] Wani, N. A., Kumar, R. & Bedi, J. Harnessing fusion modeling for enhanced breast cancer classification through interpretable artificial intelligence and in-depth explanations. *Eng. Appl. Artif. Intell.* **136**, 108939 (2024).

[34] Saharan, S., Wani, N. A., Chatterji, S., Kumar, N. & Almuhaideb, A. M. A deep learning and explainable artificial intelligence based scheme for breast cancer detection. *Sci. Rep.* **15**, 32125 (2025). 40890117 10.1038/s41598-024-80535-7 PMC12402191

[35] Mahmood, T., Saba, T., Rehman, A. & Alamri, F. S. Harnessing the power of radiomics and deep learning for improved breast cancer diagnosis with multiparametric breast mammography. *Expert Syst. Appl.* **249**, 123747 (2024).

[36] Mahmood, T. et al. Breast lesions classifications of mammographic images using a deep convolutional neural network-based approach. *PLoS ONE* **17**, e0263126 (2022). 35085352 10.1371/journal.pone.0263126 PMC8794221

[37] Wu, P., Ma, R. & Toe, T. T. Stacking-enhanced bagging ensemble learning for breast cancer classification with cnn. In *2023 3rd International Conference on Electronic Engineering (ICEEM)* 1–6 (IEEE, 2023).

[38] Singh, A. K. et al. Transforming early breast cancer detection: A deep learning approach using convolutional neural networks and advanced classification techniques. *Int. J. Comput. Intell. Syst.* **18**, 134 (2025).

[39] Mahmood, T., Li, J., Pei, Y. & Akhtar, F. An automated in-depth feature learning algorithm for breast abnormality prognosis and robust characterization from mammography images using deep transfer learning. *Biology* **10**, 859 (2021). 34571736 10.3390/biology10090859 PMC8468800

[40] Mahmood, T., Saba, T. & Rehman, A. Breast cancer diagnosis with mff-histonet: A multi-modal feature fusion network integrating cnns and quantum tensor networks. *J. Big Data* **12**, 60 (2025).

[41] Alsolami, A. S. et al. King abdulaziz university breast cancer mammogram dataset (kau-bcmd). *Data* **6**, 111 (2021).

[42] Mahmood, T., Rehman, A., Saba, T., Nadeem, L. & Bahaj, S. A. O. Recent advancements and future prospects in active deep learning for medical image segmentation and classification. *IEEE Access* **11**, 113623–113652 (2023).

[43] Sabani, A. et al. Bi-rads-based classification of mammographic soft tissue opacities using a deep convolutional neural network. *Diagnostics* **12**, 1564 (2022). 35885470 10.3390/diagnostics12071564 PMC9318280

[44] Singh, G. et al. Various image enhancement techniques: A critical review. *Int. J. Innov. Sci. Res.* **10**, 267–274 (2014).

[45] Qi, Y. *et al.* A comprehensive overview of image enhancement techniques. *Arch. Comput. Methods Eng.* 1–25 (2021).

[46] Makandar, A. & Halalli, B. Pre-processing of mammography image for early detection of breast cancer. *Int. J. Comput. Appl.* **144**, 11–15 (2016).

[47] Pisano, E. D. et al. Contrast limited adaptive histogram equalization image processing to improve the detection of simulated spiculations in dense mammograms. *J. Digit. Imaging* **11**, 193–200 (1998). 9848052 10.1007/BF03178082 PMC3453156

[48] Tripathy, S. & Swarnkar, T. Unified preprocessing and enhancement technique for mammogram images. *Procedia Comput. Sci.* **167**, 285–292 (2020).

[49] Buda, M., Maki, A. & Mazurowski, M. A. A systematic study of the class imbalance problem in convolutional neural networks. *Neural Netw.* **106**, 249–259 (2018). 30092410 10.1016/j.neunet.2018.07.011

[50] Mutlag, W. K., Ali, S. K., Aydam, Z. M. & Taher, B. H. Feature extraction methods: A review. *J. Phys. Conf. Ser.* **1591**, 012028 (2020).

[51] Medjahed, S. A. A comparative study of feature extraction methods in images classification. *Int. J. Image Graph. Signal Process.* **7**, 16 (2015).

[52] Ping Tian, D. et al. A review on image feature extraction and representation techniques. *Int. J. Multimed. Ubiquit. Eng.* **8**, 385–396 (2013).

[53] Wei-bin, L., Zhi-yuan, Z. & Wei-wei, X. Feature fusion methods in pattern classification. *J. Beijing Univ. Posts Telecommun.* **40**, 1 (2017).

[54] Mostafiz, R., Uddin, M. S., Jabin, I., Hossain, M. M. & Rahman, M. M. Automatic brain tumor detection from mri using curvelet transform and neural features. *Int. J. Ambient Comput. Intell. (IJACI)* **13**, 1–18 (2022).

[55] Chugh, G., Kumar, S. & Singh, N. Survey on machine learning and deep learning applications in breast cancer diagnosis. *Cogn. Comput.* **13**, 1451–1470 (2021).

[56] Park, S. H., Goo, J. M. & Jo, C.-H. Receiver operating characteristic (roc) curve: Practical review for radiologists. *Korean J. Radiol.* **5**, 11–18 (2004). 15064554 10.3348/kjr.2004.5.1.11 PMC2698108

[57] Ferris, M. H. *et al.* Using roc curves and auc to evaluate performance of no-reference image fusion metrics. In *2015 National Aerospace and Electronics Conference (NAECON)* 27–34 (IEEE, 2015).

[58] Eskandari, S., Eslamian, A. & Cheng, Q. Comparative analysis of transfer learning models for breast cancer classification. *arXiv preprint* arXiv:2408.16859 (2024).

