# Supplementary material for: Different BI-RADS breast cancer diagnosis using MobileNetV1 and vision transformer based on explainable artificial intelligence (XAI)
Source: Sci Rep. 2026 Feb 17;16:7190. doi: 10.1038/s41598-026-37199-2 (PMC12920763; doi:10.1038/s41598-026-37199-2)
Supplement: Supplementary file 1 — Supplementary Information. [file 41598_2026_37199_MOESM1_ESM.pdf]

Supplementary Material: Mathematical Formulations

S1. Global Histogram Equalization

$$p'(i) = \frac{M-1}{N}\sum_{j=0}^{i} p(j)$$

S2. CLAHE Clipping

$$h'_k(i) = \begin{cases} h_k(i), & \text{if } h_k(i) \leq \text{ClipLimit} \\ \text{ClipLimit}, & \text{if } h_k(i) > \text{ClipLimit} \end{cases}$$

S3. CLAHE Mapping Function

$$p'_k(i) = \frac{M-1}{N_k}\sum_{j=0}^{i} h'_k(j)$$

S4. Image Normalization

$$I_{\text{norm}} = \frac{I(x,y) - I_{\text{mean}}}{I_{\text{sdv}}}$$

S3. Mathematical Formulations of Feature Extraction

1. Depthwise Separable Convolutions in MobileNetV1

Depthwise Convolution:

$$Y_{\text{dw}}(i,j,c) = \sum_{m=1}^{k}\sum_{n=1}^{k} X(i+m-1, j+n-1, c) \cdot F(m,n,c)$$

Pointwise Convolution:

$$Y(i,j,c_{\text{out}}) = \sum_{c=1}^{C_{\text{in}}} Y_{\text{dw}}(i,j,c) \cdot F_p(1,1,c,c_{\text{out}})$$

Residual Connection (optional):

$$Y(i,j,c_{\text{out}}) = F(X(i,j,\cdot)) + Y_{\text{dw}}(i,j,c_{\text{out}})$$

2. Vision Transformer (ViT)

Patch Embedding:

$$z_p = W_e \cdot x_p + b_e$$

Positional Encoding:

$$Z = [z_{\text{class}}; z_1 + p_1; z_2 + p_2; \dots ; z_N + p_N]$$

Self-Attention:

$$\text{Attention}(Q,K,V) = \text{softmax}\left(\frac{QK^\top}{\sqrt{d}}\right)V$$

Feed-Forward Network (FFN) and Residuals:

$$z' = \text{LayerNorm}(z + \text{Attention}(Q,K,V)), z'' = \text{LayerNorm}(z' + \text{FFN}(z'))$$

S4. Fine-Tuning, Feature Fusion, and Classification

1. Feature-Level Fusion

$$F_{\text{final}} = [F_{\text{MN}} + F_{\text{ViT}}]$$

2. Bagging Ensemble with Logistic Regression

$$F(x) = \arg\max_{y} \sum_{i=1}^{B} \mathbf{1}\{ f_i(x) = y\}$$
